# Time-series transcriptome provides insights into the gene regulation network involved in the volatile terpenoid metabolism during the flower development of lavender

**DOI:** 10.1186/s12870-019-1908-6

**Published:** 2019-07-15

**Authors:** Hui Li, Jingrui Li, Yanmei Dong, Haiping Hao, Zhengyi Ling, Hongtong Bai, Huafang Wang, Hongxia Cui, Lei Shi

**Affiliations:** 1 Key Laboratory of Plant Resources and Beijing Botanical Garden, Institute of Botany, Chinese Academy of Sciences, Xiangshan, Beijing, 100093 China; 2 University of Chinese Academy of Sciences, Beijing, 100049 China; 3 College of Biological Sciences and Biotechnology, National Engineering Laboratory for Tree Breeding, Beijing Forestry University, Beijing, 100083 China

**Keywords:** *Lavandula angustifolia* ‘JX-2’, Pollinator, Herbivore, Volatile terpenoids, Flowering, Network analysis

## Abstract

**Background:**

Essential oils (EOs) of *Lavandula angustifolia*, mainly consist of monoterpenoids and sesquiterpenoids, are of great commercial value. The multi-flower spiciform thyrse of lavender not only determines the output of EOs but also reflects an environmental adaption strategy. With the flower development and blossom in turn, the fluctuation of the volatile terpenoids displayed a regular change at each axis. However, the molecular mechanism underlying the regulation of volatile terpenoids during the process of flowering is poorly understood in lavender. Here, we combine metabolite and RNA-Seq analyses of flowers of five developmental stages at first- and second-axis (FFDSFSA) and initial flower bud (FB0) to discover the active terpenoid biosynthesis as well as flowering-related genes.

**Results:**

A total of 56 mono- and sesquiterpenoids were identified in the EOs of *L. angustifolia* ‘JX-2’. FB0’ EO consists of 55 compounds and the two highest compounds, *β*-trans-ocimene (20.57%) and (+)-R-limonene (17.00%), can get rid of 74.71 and 78.41% aphids in Y-tube olfactometer experiments, respectively. With sequential and successive blossoms, temporally regulated volatiles were linked to pollinator attraction in field and olfaction bioassays. In three characteristic compounds of FFDSFSA’ EOs, linalyl acetate (72.73%) and lavandulyl acetate (72.09%) attracted more bees than linalool (45.35%). Many transcripts related to flowering time and volatile terpenoid metabolism expressed differently during the flower development. Similar metabolic and transcriptomic profiles were observed when florets from the two axes were maintained at the same maturity grade. Besides both compounds and differentially expressed genes were rich in FB0, most volatile compounds were significantly correlated with FB0-specific gene module. Most key regulators related to flowering and terpenoid metabolism were interconnected in the subnetwork of FB0-specific module, suggesting the cross-talk between the two biological processes to some degree.

**Conclusions:**

Characteristic compounds and gene expression profile of FB0 exhibit ecological value in pest control. The precise control of each-axis flowering and regular emissions at transcriptional and metabolic level are important to pollinators attraction for lavender. Our study sheds new light on lavender maximizes its fitness from “gene-volatile terpenoid-insect” three layers.

**Electronic supplementary material:**

The online version of this article (10.1186/s12870-019-1908-6) contains supplementary material, which is available to authorized users.

## Background

The family Labiatae is distributed worldwide and includes over 250 genera and approximately 7000 species [1]. This family is known for its fine ornamental or culinary herbs such as basil, lavender, mint, oregano, rosemary, sage and thyme, and is an abundance source of essential oils (EOs). Plants of the *Lavandula* genus, especially *L. angustifolia*, *L. latifolia* and their natural sterile hybrid *L*. × *intermedia,* are now cultivated worldwide for their EOs, which are widely used in perfumes, cosmetics, pharmaceuticals and, more recently, in aromatherapy products [2]. *L. angustifolia* subsp. angustifolia, distributed in southern France and extending into the Italian Alps to the east and to Calabria in southern Italy, occurs naturally in very arid habitats from (250-) 500 to 1800 (− 2000) m on calcareous soils [3]. Inflorescence architecture is among the most important characteristics of adaption and provides the very basic structural foundation that determines the maximum number of sites available for seed production and even the genetic diversity of the progenies [4]. *L. angustifolia* is a cincinnus (containing 3~9 florets) with bisymmetric cymes divaricating 2~3 times, resulting in spiral or scorpoid forms. This inflorescence architecture is an efficient way to pack more flowers into a dense spike and, more importantly, can produce new flowers continuously over a period of at least several weeks. Although many florets of various developmental stages (from immature to fade) are scattered on one rachis, the blooming order of these florets is finely tuned: the two symmetric terminal florets open first from the base to the top whorl, followed by the four florets on the second axis that developed from each first axis (Additional file 19: Video S1). This blooming sequence helps to maximize out-crossing by increasing the potential number of different male parents, filling up for the less outputs of four one-seeded nutlets in each flower and the resulting heterogeneous patterns enhance the capacity to adapt to changes in the environment. Previous studies paid attention to the individual floret at a random position of the inflorescences or to whole inflorescences with different bloom ratios during the flowering period, but chemical and molecular analyses of different-axis florets at the same whorl were neglected [5, 6], as was the ecological significance behind this blooming sequence.

The chemical components of lavender and lavandin EOs are characterized by the presence of monoterpenoids (e.g. linalool and linalyl acetate) and sesquiterpenoids (e.g. caryophyllene and bergamotene) and other irregular types. The lineage-specific terpenoids, which have arisen throughout the evolution of green plants, have generally been postulated to play an important role in mediating ecological interactions between plants with a diverse array of visitors, including pollinators, herbivores, natural enemies and pathogens, ensuring the plants’ reproductive and evolutionary success [7, 8]. Extensive evidence has indicated that the spatiotemporal expression of terpene synthase (TPS) is correlated with the biosynthesis and emission of volatile terpenoids, such as the *linalool synthase* (*LINS*) and *limonene synthase* (*LIMS*) reported in lavender, indicating that this regulation may occur, at least in part, at the transcriptional level [5, 6].

Lavender inflorescence is a typical model for studying the regulation of terpenoid synthesis at the molecular, cellular and ecological levels [9]; however, there is still almost no comprehensive understanding of terpenoid metabolism via network-focused rather than individual gene/protein-focused strategies in lavender. A nucleotide search of GenBank revealed only 15 lavender-derived sequences related to terpenoid biosynthesis (Additional file 12: Table S1), which is quite insufficient compared to the pace of identification of volatile compounds in lavender. Moreover, previous studies have described the expression patterns of only a few TPSs with flower development [5]. Furthermore, the orchestrated formation of various terpenoids is not only a function of biosynthetic enzymes, but also requires the involvement of the poorly understood terpenoid modification enzymes [e.g., cytochrome P450 monooxidase (CYP450s)], transcription factors (TFs) and terpenoid transporters.

As a powerful modern genetic research tool, next-generation sequencing techniques have been widely used to analyse many non-model organisms due to their low cost and high output. Many computational methodologies, such as weighted gene co-expression network analysis (WGCNA), are designed to provide insight at the system level and have been applied to high-throughput RNA-Seq datasets to detect molecular communication [10]. The clustering of genes in a co-expressed group indicate close regulatory associations between them, thus enabling inference of the biological function of unknown genes by ‘guilt by association’ with well-characterized ones. It is particularly noteworthy that co-expressed gene groups can be combined with metabolite datasets, and this strategy can detect genes (including both transcription factor and enzyme genes) that are highly correlated with specific chemicals [8]. A number of studies have successfully applied WGCNA to microarray and metabolite data to develop metabolite-specific gene atlases, for plants such as *Thesium chinense* [11], *Vitis vinifera* [12] and *Anthurium amnicola* [13].

*L. angustifolia* occupies a top-tier position in the aromatic plant list from both economic and ecological perspectives and is regarded as a genomic model for studying terpenoid metabolism. However, to our knowledge, there are no global transcriptomic and metabolic analyses related to its flower development. A main objective of the present work was to examine the ecological implications of sequential and successive blossoms of lavender inflorescences and explore how lavenders orchestrate flowering time and the emissions of diverse terpenoids. Our results pave the way for a detailed understanding of the regulation of mono- and sesquiterpenoid synthesis in lavender and shed new light on the ecological and genetic flexibility of lavender inflorescences.

## Results

### Volatile terpenoid profiles of individual florets at different developmental stages

**Fig. 1:**
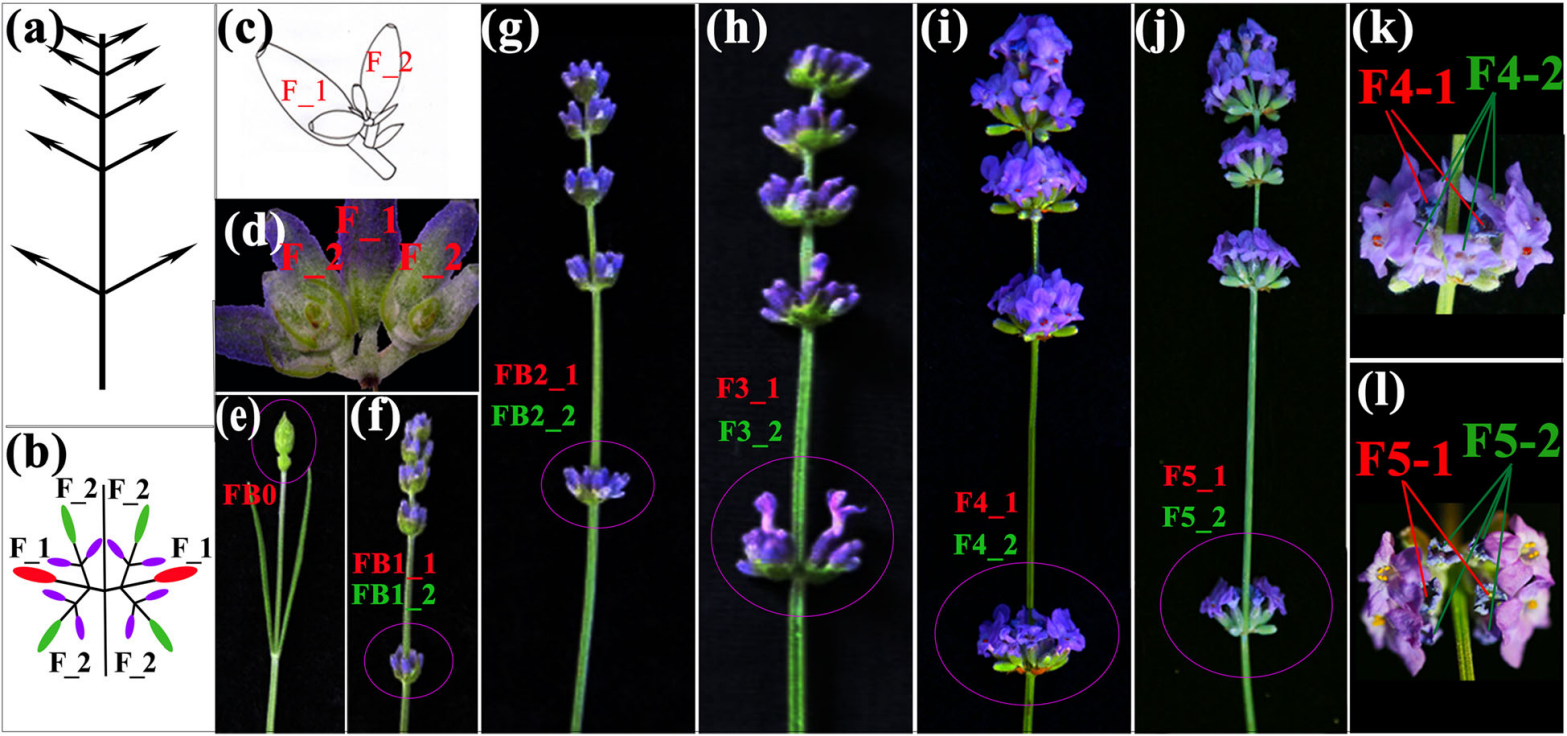
The morphology of flower at various developmental stages in lavender. **a**-**d** Schematic representations of *L. angustifolia* ‘JX-2’ inflorescence. Inflorescence with five-whorl flower was selected (**a**). **b** a pair of multi-flowered cyme. Red ovals represent first-axis florets, which were indicated ‘F_1’ in (**b**-**d**), and green ovals represent second-axis florets attached to first axis, which were indicated ‘F_2’ in (**b**-**d**). **e**-**l** initial bud (**e**) as well as first- and second-axis flowers at remote whorl (circled by magenta) of five maturity stages were used for analysis. The florets of first- or second-axis at fourth and fifth developmental stage were indicated by red or green lines, respectively (**k**, **l**)

**Fig. 2:**
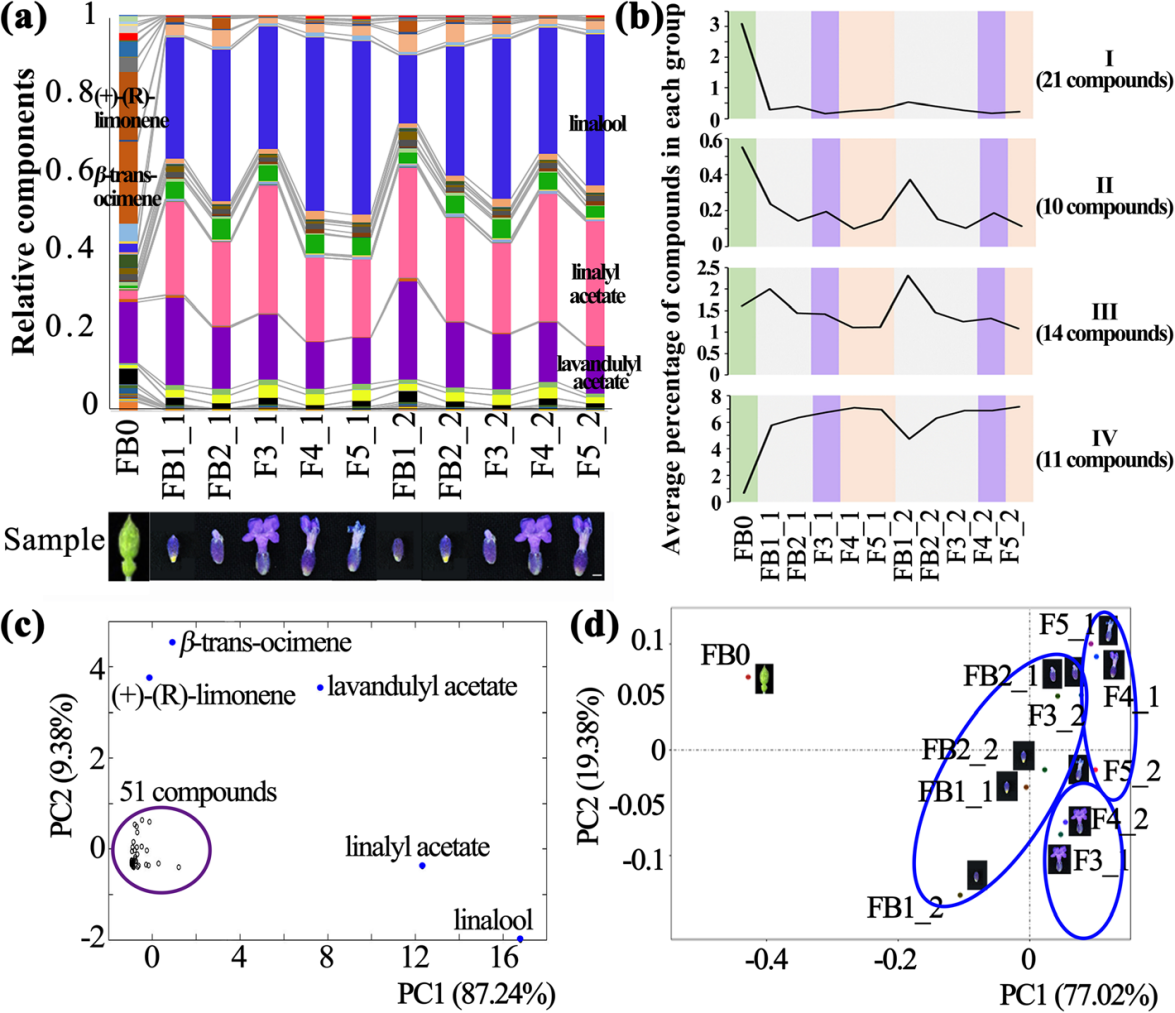
Volatile terpenoids of flower at various developmental stages in lavender. **a** The relative percentage of 56 terpenoid compounds at 11 samples. The morphology of samples was placed in the below. **b** The mean contents of compounds with development in each group. **c** PCA of 56 compounds in 11 samples. **d** PCA of 11 samples based on 56 compounds

We selected the remote-whorl florets of the first and second axes at five developmental stages and the initial flower buds (a total of 11 types of flower samples, see Methods) for EO extraction (Fig. 1). A total of 34 monoterpenoids and 22 sesquiterpenoids were detected in at least one of the 11 lavender samples by GC-MS analysis. The content of each terpenoid was presented as a percentage of the total volatiles, and the 56 compounds added up to 99.99% of the total volatiles (Additional file 13: Table S2). The results showed that florets at different developmental stages had distinct volatile profiles, especially the EO composition of FB0 compared to the flowers of the five developmental stages of the first and second axes (FFDSFSA) (Fig. 2a; Additional file 1: Figure S1). A higher proportion of (+)-(R)-limonene (17.00%), *β*-trans-ocimene (20.57%), o-cymene (3.72%) and 3-carene (3.83%) and a lower proportion of linalool (2.18%), linalyl acetate (0.73%) and α-terpineol (0.62%) were characteristic of volatile extracted from FB0. With the expansion of the initial bud into five-whorl purple flowers, the main components of the florets became linalool (17.06~43.72%, indicated by blue blocks), linalyl acetate (19.35~32.24%, indicated by magenta blocks) and lavandulyl acetate (11.60~24.46%, indicated by purple blocks) with fluctuations at FFDSFSA (Fig. 2a, Additional file 13: Table S2). We also normalized the contents of volatiles using the z-score, and 56 compounds detected during different stages of flower development were clustered into four groups (Additional file 2: Figure S2). In groups I and II, 31 compounds exhibited the highest accumulation at FB0. Meanwhile, the average contents of 10 compounds in group II showed a peak at anthesis. The 14 compounds in group III accumulated in both FB0 and unopened florets and decreased with the flower maturity, while the 11 compounds in group IV increased with maturity, showing the lowest accumulation in FB0 (Fig. 2b).

When principal component analysis (PCA) applied in the 56 compounds, we found that (+)-(R)-limonene and *β*-trans-ocimene, which are the two highest compounds of FB0’ EOs, and linalool, linalyl acetate and lavandulyl acetate, which are the main components of FFDSFSAs’ EOs, were distinct from the rest compounds (Fig. 2c). Moreover, the volatile composition of individual floret from the first or second axis was significantly influenced by morphological maturity. Obviously, the contents of linalyl acetate and lavandulyl acetate elevated, but the content of linalool decreased when the florets were blooming regardless of whether they were on the first or second axis (F3_1 and F4_2). During the flower fading stages (F4_1, F5_1 and F5_2), the content of linalool increased. As confirmed by PCA of the profiling data for these 56 volatiles from the 11 flower samples, the unopened flower buds, blooming flowers and fading flowers of the two axes were well clustered and the area of distribution of FB0 differed more significantly from FFDSFSA (Fig. 2d). In summary, oscillations in volatile accumulation were closely linked to the developmental stage of the individual floret.

### Observation of floral visitors and olfaction bioassays

**Fig. 3:**
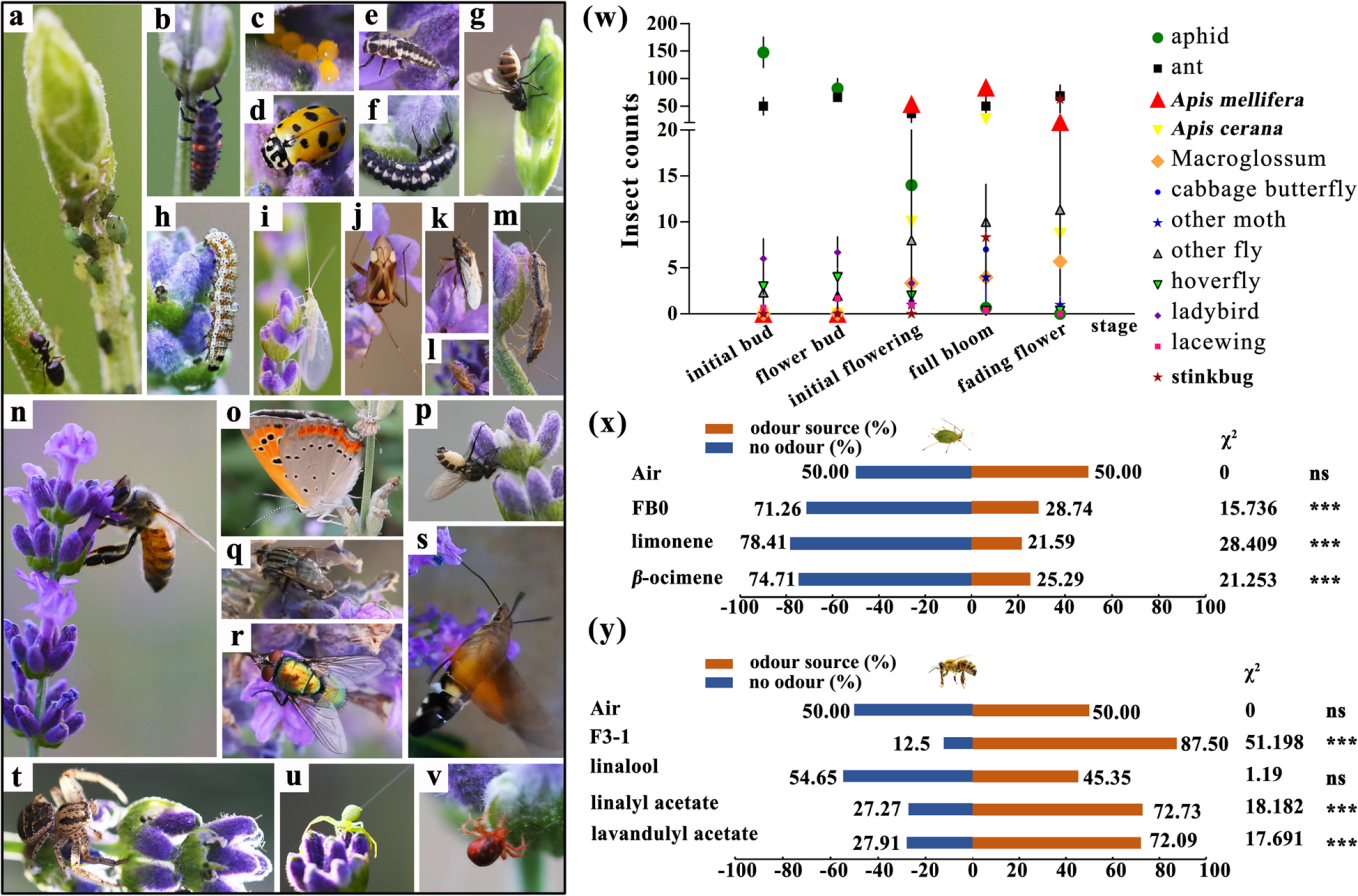
The field observation and laboratory experiment of herbivores, pollinators or predators. **a**-**v** Various insects and spiders visit lavender inflorescence of different developmental stages, including aphid (**a**), ladybird (**b**-**f**), lacewing (**h**, **i**), stinkbug (**j**-**m**), *Apis mellifera* (**n**), syrphid (**g**, **p**), *Macroglossum pynhostictum* (**s**), *Pieris rapae* (**o**), *Sarcophaga spp.* (**q**), *Calliphora vicina* (**r**) and spiders (**t**-**v**). **w** The field statistics of insects of different developmental inflorescences. **x**, **y** The preferring selection to volatiles of aphids or bees in Y-tube containing standards diluted specific proportion by white oil and white oil (control). Asterisks indicate significant differences of these dual choice detected by χ^2^ test (ns, non-significant difference *P* > 0.05, * *P* < 0.05, ** *P* < 0.01 *** *P* < 0.001)

Our preliminary field investigation found that few pollinators showed up before blossoming, while lavender was often attacked heavily by aphids (herbivore) at FB0 during spring in Beijing (Fig. 3a, w). Then the number of aphids is diminishing with flower development and bare of them was left when lavender blooms (Fig. 3w). Some other herbivores, like *Tetranychus cinnbarinus* (Fig. 3v)*,* stinkbugs (Fig. 3j-m) were also observed in lavender. Simultaneously, we observed eggs, larva and imago of some predatory species, including ladybirds, lacewings, spiders and hoverflys on the inflorescences of lavender (Fig. 3b-i, t, u). When the first pair of florets bloomed, bees (including *Apis mellifera* and *A. cerana*) began to visit the lavender plants repeatedly (Fig. 3n). As the percentage of bloom increased, a growing bee population was observed (Fig. 3w). In addition, the blooming flowers attracted many other pollinators, such as *Macroglossum pynhostictum*, *Pieris rapae*, *Sarcophaga* spp. and *Calliphora vicina* (Fig. 3o-s).

In the Y-tube olfactometer bioassay, 71.26% aphids kept away from the FB0’ EOs and chose the orientation without ordour (Fig. 3x). When aphids were given the choice between no odour and the odours of the two compounds, they significantly preferred no odour over the single *β*-trans-ocimene (diluted to 20.57%) or (+)-(R)-limonene (diluted to 17.00%), which was diluted to the same proportion as in FB0, suggesting the phobotaxis of these compounds to aphids (Fig. 3x). For assays of bees, the lavender oil extracted from blooming flowers (F3_1) attracted significantly more bees (87.50%) than a single standard substance (linalyl acetate, 72.73%; lavandulyl acetate, 72.09%; linalool, 45.35%). Linalyl acetate or lavandulyl acetate alone attracted more bees than linalool (Fig. 3y). The increased contents of linalyl acetate and lavandulyl acetate at the blooming stage and the greater attraction of bees implied that the linalyl acetate and lavandulyl acetate may contribute more to the attraction of pollinators, than linalool in lavender.

### RNA sequencing and different expression genes (DEGs) analysis

**Fig. 4:**
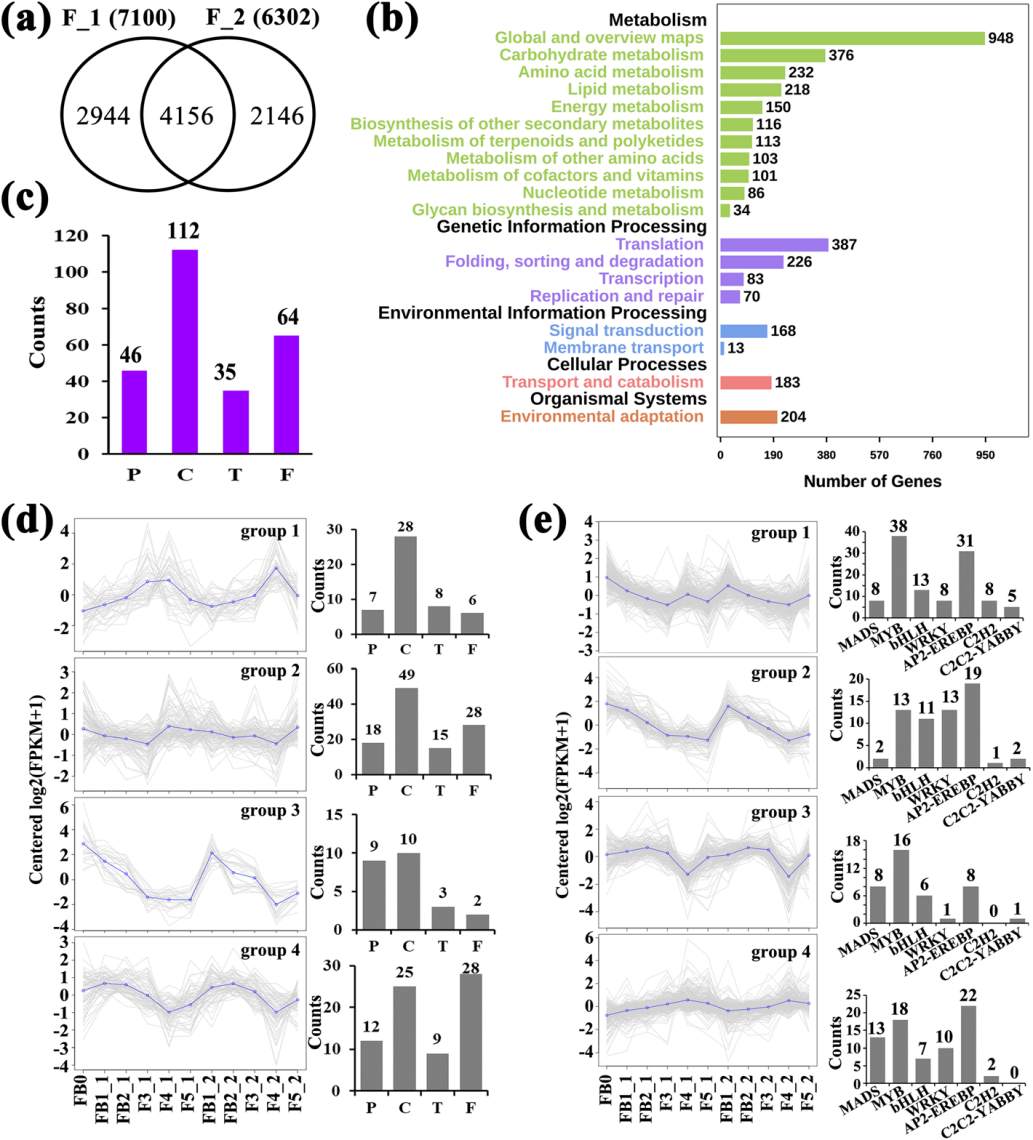
DEGs analysis. **a** The overlapping of common genes from the two axes (F_1 and F_2). Total 9246 genes expressed differentially in flower development of first- or second-axis. **b** KEGG pathway classification map of 9246 DEGs. **c** The DEGs counts related to four types genes (P, C, T and F). **d** The clustering of co-expressed four types genes. The number of four types genes was shown on the right. **e** The clustering of TFs. The number of terpenoid and flowering-related TFs was shown on the right

RNA sequencing was performed to obtain the global transcriptomic dynamics in FFDSFSA and FB0. Total 159,337 unigenes (≥200 bp) could be detected with an N50 length of 1130 bp and 78,464 unigenes were successfully annotated to at least one database (Additional files 3, 4, 14, 15: Figures S3, S4; Tables S3, S4). Comparing FFDSFSA with FB0 resulted in 15,212 (FB1_1 vs FB0), 19,887 (FB2_1 vs FB0), 24,424 (F3_1 vs FB0), 25,305 (F4_1 vs FB0), 23,929 (F5_1 vs FB0), 15,150 (FB1_2 vs FB0), 17,914 (FB2_2 vs FB0), 22,408 (F3_2 vs FB0), 28,289 (F4_2 vs FB0), 22,405 (F5_2 vs FB0) different expression genes (DEGs), respectively (Additional file 5: Figure S5). These data suggested that a dramatic alteration of gene expression would result in significant changes in the contents of volatile terpenoids. We identified 7100 and 6302 DEGs shared by FFDSFSA and FB0, respectively (Additional file 6: Figure S6). Finally, in-depth analysis was further carried out on 9246 DEGs, including 4156 genes expressed differentially in the two axes, with 2944 and 2164 DEGs specifically in the first and second axes, respectively (Fig. 4a). Enrichment analysis showed 113 genes involved in “metabolism of terpenoids and polyketides” pathway (Fig. 4b).

### The temporal expression patterns of flowering- and terpenoid metabolism-related genes during individual flower development

Given the ecological importance of flower opening time and floral scent emissions, the orthologous genes related to those two coordinated traits were manually selected. Differentially expressed transcripts encoding proteins with presumed (homology-based) functions in the biosynthesis, modification and transport of terpenoids as well as flowering were identified, including 46 genes in the terpenoid synthesis pathway (P), 35 terpenoid transporters (T), 112 cytochrome P450 hydroxylases (C) and 64 flowering-associated genes (F) (Fig. 4c; Additional files 8, 16, 17: Figure S8, Table S5, 6). These four types of genes (P, T, C and F) can be classified into four groups with obvious stage-specific expression trends by k-means. Similar expression trends were observed at the same developmental stage between the first- and second-axis flowers. The expression of genes in group 1 (49 genes) and group 4 (74 genes) peaked at flowering time and flower budding, respectively. Twenty-four genes clustered in group 3 showed the strongest expression at FB0, while 110 genes in group 2 showed the highest expression level when the flower started to wither (Fig. 4d). We also found that the genes of four types can be detected in each group, suggesting that these genes may participate in the same biological processes. We also identified a total of 807 (approximately 8.7% to 9246 DEGs) TFs belonging to 69 families among 9246 DEGs (Additional file 7: Figure S7), and these TFs were classified into four co-expression groups (Fig. 4e). The TFs in groups 1–3 were decreased with maturity, while TFs in group 4 exhibited an escalating trend. The terpenoid- (*MYB*, *bHLH*, *WRKY*, *C2H2*, *C2C2-YABBY*, *AP2-EREBP*) and flowering-related (*MADS*) TFs in each group were specially counted (Fig. 4e).

**Fig. 5:**
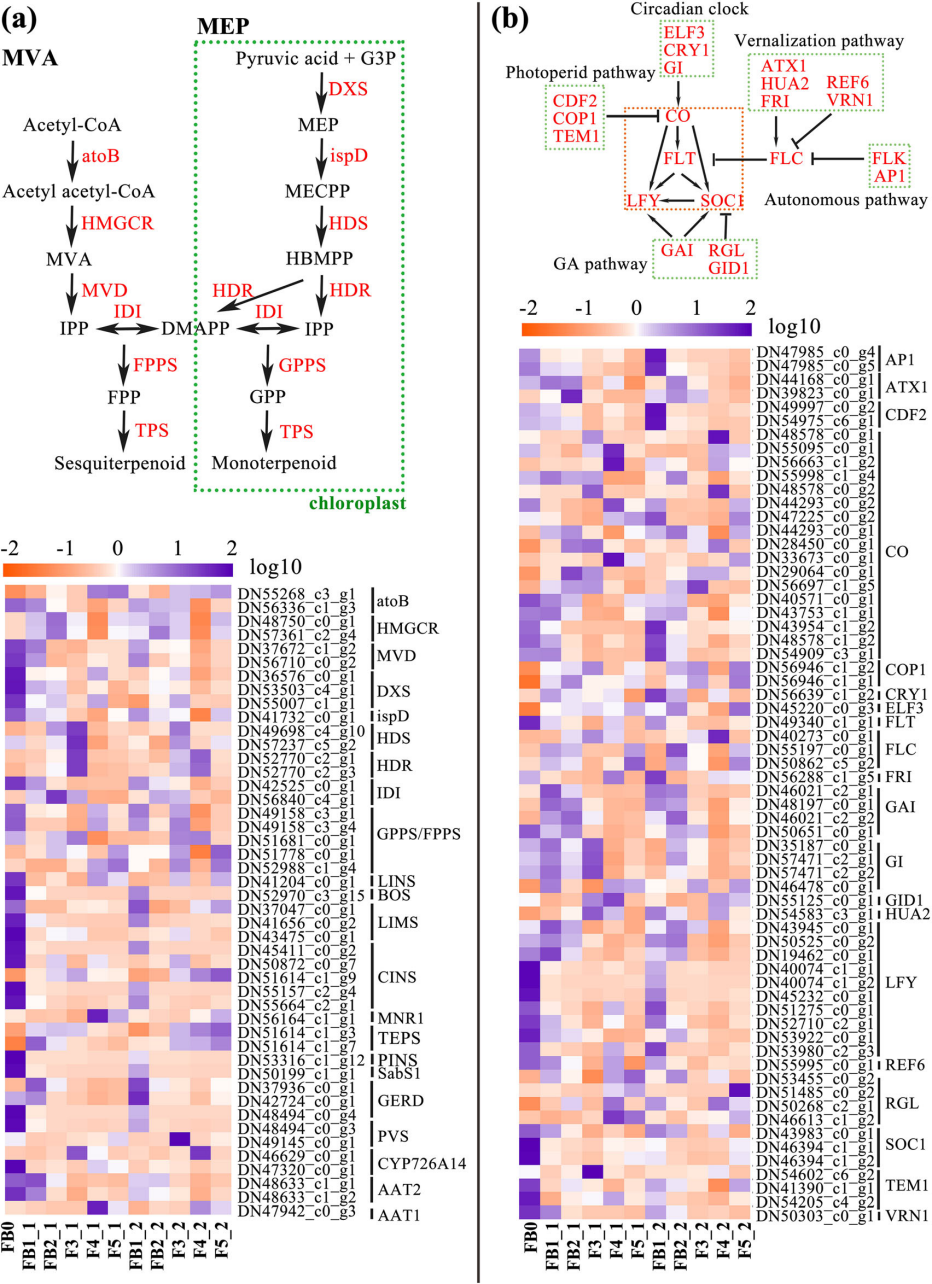
Heatmap and schematic diagram for genes related to terpenoid biosynthesis (**a**) and flowering time (**b**). atoB, acetyl-CoA acetyltransferase; HMGCR, 3-hydroxy-3-methylglutaryl-coenzyme A reductase; ispD, 2-C-methyl-D-erythritol 4-phosphate cytidylyltransferase; CINS, 1,8-cineole synthase; MNR1, (+)-neomenthol dehydrogenase; PINS, pinene synthase; PVS, premnaspirodiene oxygenase. ATX1, copper transport protein; CDF2, cyclic dof factor 2; COP1, E3 ubiquitin-protein ligase; CRY1, cryptochrome-1; ELF3, early flowering 3; FLC, flowering locus C; FRI, frigida; GAI, DELLA protein; GI, gigantea; GID1, gibberellin receptor; RGL, DELLA protein; REF6, lysine-specific demethylase; HUA2, glutathione peroxidase; TEM1, RAV-like factor; VRN3, B3 domain-containing transcription factor

Plant terpenes are synthesized in the plastids through the mevalonic acid (MEP) and in the cytosol through the methylerythritol phosphate (MVA) pathway, followed by condensation reaction catalyzed by geranyl diphosphate synthase (GPPS) or farnesyl diphosphate synthase (FPPS) to form basic precursors of monoterpenoids (C10) and sesquiterpenoids (C15), respectively. And various TPSs catalyze a key biosynthetic step, leading to the production of tens of thousands of terpenoid compounds (Fig. 5a). In all, 46 unigenes related to 23 enzymes for monoterpenoids and sesquiterpenoids biosynthesis were obtained in this transcriptome database. Most of the P type genes were highly expressed in the flower bud, especially in the initial bud, such as *1-deoxy-D-xylulose-5-phosphate synthase* (*DXS*), *mevalonate diphosphate decarboxylase* (*MVD*), *isopentenyl-diphosphate delta isomerase* (*IDI*), while *(E)-4-hydroxy-3-methylbut-2-enyl-diphosphate synthase* (*HDS*) and *4-hydroxy-3-methylbut-2-en-1-yl diphosphate reductase* (*HDR*) were upregulated at the early stage of bloom (Fig. 5a). The transcript levels of *LIMS*, *ocimene synthase* (*BOS*), *germacrene D synthase* (*GRED*) and putative *sabinene synthase* (*SabS1*) were highest at FB0 and then downregulated, corresponding with the contents of the products they catalyse. In contrast, the expression level of putative *α-terpineol synthase* (*TEPS*) was significantly downregulated at FB0, and the content of *α*-terpineol was lowest at this stage. Additionally, since lavandulol served as the preferred substrate for alcohol acetyltransferase (AAT) to synthesize lavandulyl acetate in vitro, we found that the expression of *AAT2* was upregulated before blossom, which coincided with the change in lavandulyl acetate content (Fig. 5a). Five genes, including *flowering locus T* (*FLT*), *suppressor of overexpression of constans 1* (*SOC1*), *apetala1* (*AP1*), *leafy* (*LFY*) and *constans* (*CO*), are main floral integrator of five pathways (circadian clock, photoperiod, gibberellins, vernalization and autonomous pathways) (Fig. 5b). Most of the floral integrator genes were expressed at high levels before flowering. Candidate genes in the five pathways showed variable expression patterns, suggesting an intricate regulatory network of these genes governing floral opening in lavender (Fig. 5b).

### Co-expression network analysis

**Fig. 6:**
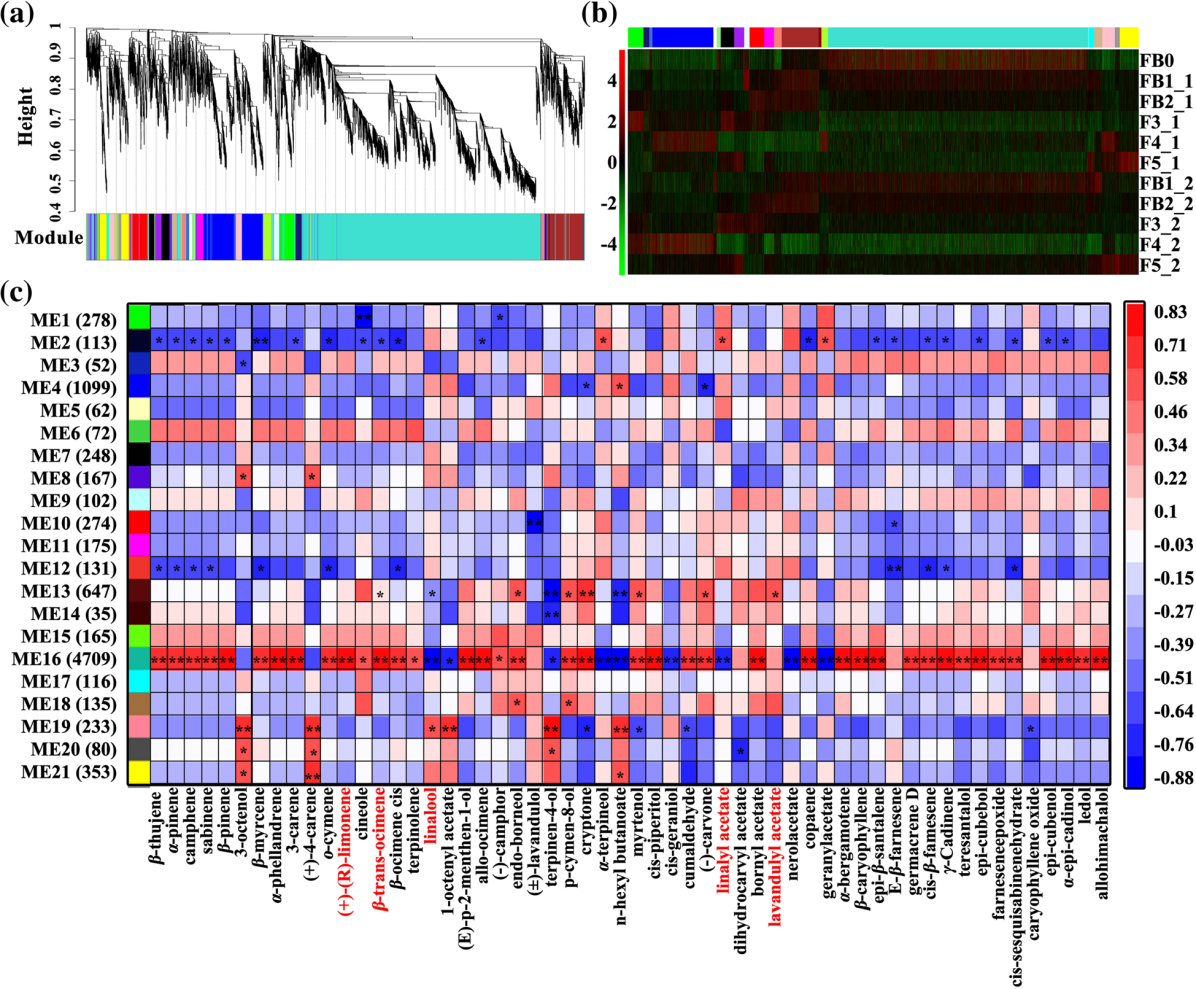
WGCNA of 9246 DEGs at different developmental stages of lavender flower. **a** Hierarchical cluster tree showing 22 modules of co-expressed genes. Each of the 9246 DEGs is represented by a leaf of the tree and major tree branches constitute 22 modules, labeled with different colors at the lower panel. Note that module ‘Grey’ is for unassigned genes. **b** Expression heatmap of genes clustered in 21 modules (exclude module ‘Grey’). **c** The correlations between 56 volatile terpenoids and modules. Each row represents a module and each column represents a terpenoid. The color of each block at the row–column intersection indicates the correlation coefficient: red for high positive correlation and blue for high negative correlation, with a scale shown on the right of the panel. The number of DEGs involved in each module is presented in parentheses in the left panel. Asterisks indicate significant correlations (*P < 0.05, **P < 0.01)

To capture comprehensive transcriptome changes during the development of individual floret located on both axes, we built weighted gene co-expression networks to classify 9246 DEGs. Highly interconnected genes were clustered in the same module and we ultimately obtained 21 distinct modules (M1-M21, excluding module grey) shown in the dendrogram (Fig. 6a). Obviously, the gene expression profiles of first- and second-axis flowers had analogous rhythms and showed strong temporal expression patterns across ontogenesis (from immature to fade). Moreover, M16 contained a large number of genes (4709) with the strongest expression level at FB0 (Fig. 6b). The module eigengenes (MEs) can be considered to be representative of the gene expression profile in a given module [14]. All 21 modules were clustered into six categories based on MEs (Additional file 9: Figure S9). Correspondingly, the relationship between modules and sample types was also recognized. The MEs of the 21 distinct modules were each correlated with distinct sample types due to their stage-specific expression profiles. Modules assembled in one group had similar sample traits. In addition, 16 out of 21 co-expression modules were significantly correlated with a single sample type (*P* < 0.05). The co-expression network approach successfully incorporated developmental stage information to describe ‘modules’ based on their expression patterns, providing a more integrated view of the stage-specific transcriptome.

The module-volatile terpenoids relationship revealed that each terpenoid was significantly relevant to at least one module (Fig. 6c). Remarkably, a large portion of terpenoids (40 out of 56 compounds), most of which showed peak accumulation at FB0 [such as (+)-(R)-limonene, *β*-trans-ocimene, o-cymene and 3-carene], exhibited strongly positive correlation with M16 (which was positively correlated with FB0). Nine compounds, most of which showed low accumulation at FB0 (such as linalool, linalyl acetate, terpinen-4-ol and α-terpineol), were negatively correlated with M16. Moreover, 24 and 11 compounds showed significant correlation with M2 and M12 (which were negatively correlated with FB0), respectively. Interestingly, the compounds that were negatively correlated with M2 and M12 were positively correlated with M16 and vice versa. Eleven compounds were correlated with one or more fading stage-related modules (M19-M21). In M1 and M2, the gene expression level appeared to be elevated at anthesis of both the first and second axes. Cineole and (−)-camphor showed negative correlation with M1, while geranyl acetate, linalyl acetate and α-terpineol showed positive correlation with M2. Then, we focused on the relationship between the modules and the levels of three main compounds: M16, M13 and M19 were highly correlated with linalool; M16 and M2 were negatively correlated with linalyl acetate; and M13 was positively correlated with lavandulyl acetate.

### Identification of candidate genes related to terpenoid metabolism during flower development

**Fig. 7:**
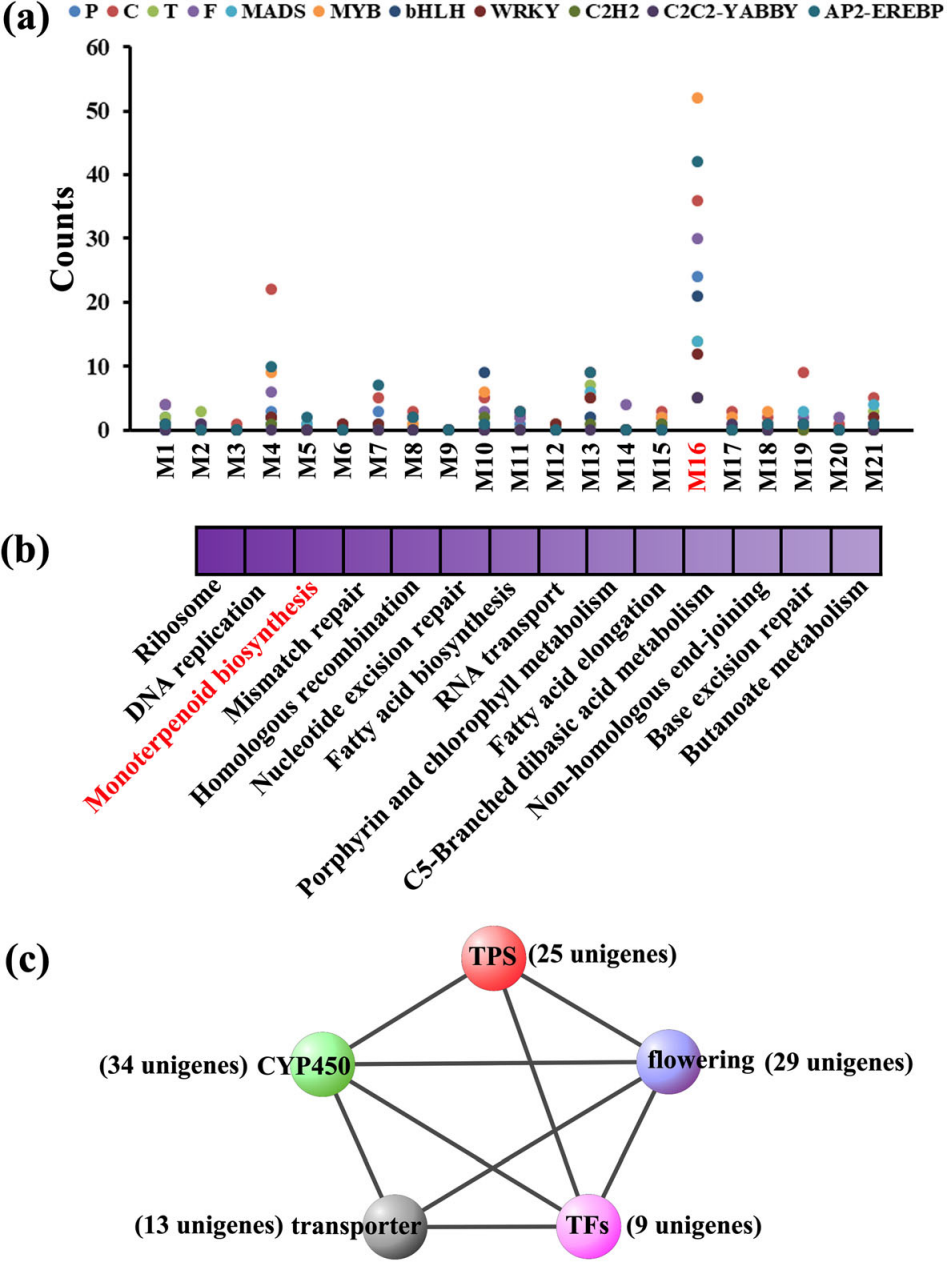
Candidate genes involved in flowering and terpenoid metabolism of 21 co-expression modules as well as the functional enrichment and subnetwork of M16. **a** Number of flowering-related genes (F and MADS) and terpenoid metabolism-related genes (P, C, T, *MYB*, *bHLH*, *WRKY*, *C2H2-YABBY* and *AP2-EREBP*) in each co-expression module. **b** Top 20 KEGG pathway enriched in M16. **c** Subnetwork visualization of M16. Nodes of different color represent groups of different type genes. The edges between nodes represent correlation of them

**Fig. 8:**
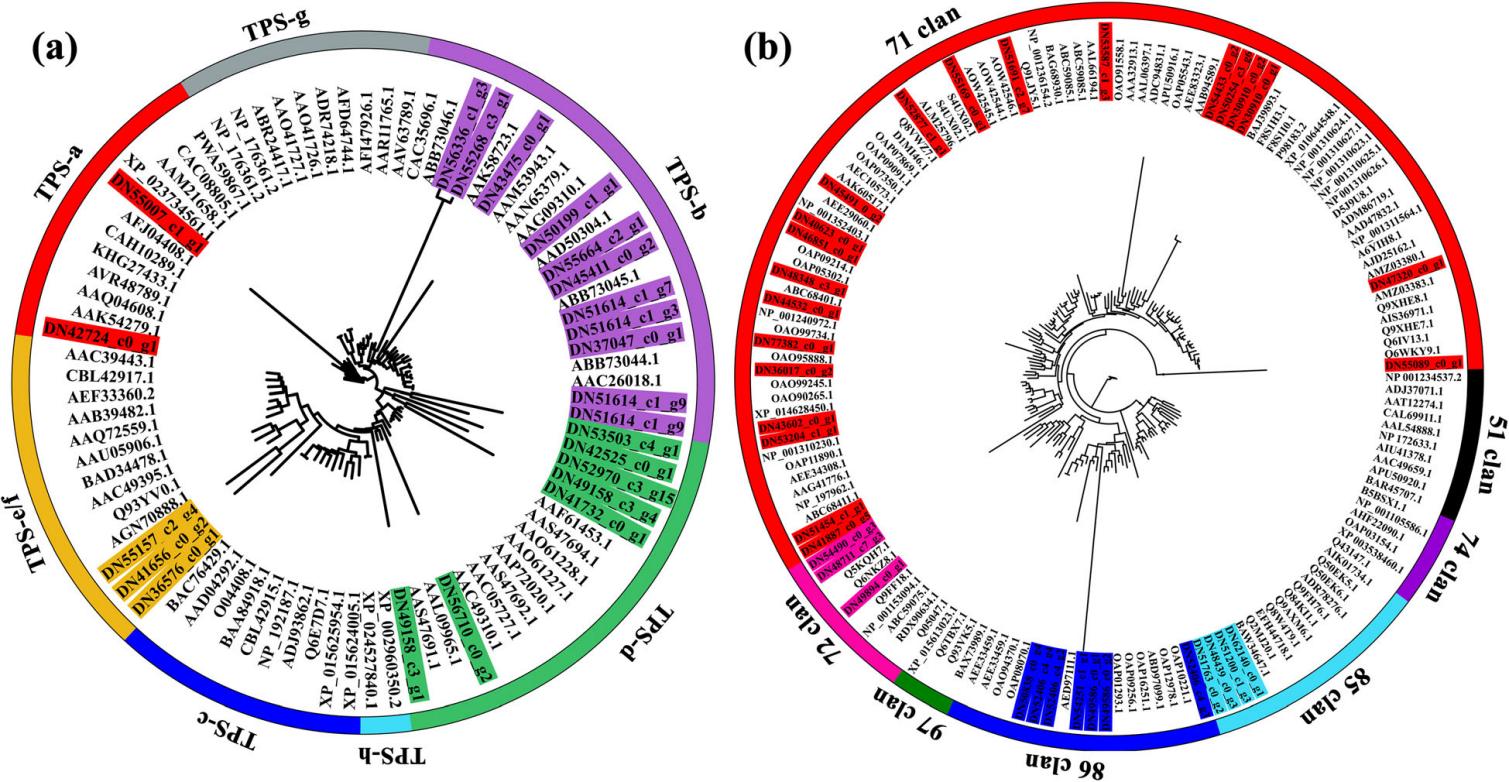
Phylogenetic analysis of TPS and CYP450 families of M16 based on deduced amino acid sequence. The phylogenetic tree was constructed using RAXMLHPC2_TGB tool with the maximum likelihood method and displayed using FigTree v1.4.3. **a** TPS proteins are clustered into seven subfamilies. **b** Proteins of CYP450 family are grouped into seven clans. Putative proteins of ‘JX-2’ are highlighted with corresponding subfamily or clan color

To validate the biological relevance of every module, we analysed the top 20 enriched pathways of each module by alignment of genes in the KEGG database. Half of all modules, including M1, M6–8, M11–13, M15, M16, M18 and M19 covered pathways associated with ‘terpenoid backbone biosynthesis’, ‘monoterpenoid biosynthesis’, sesquiterpenoid and triterpenoid biosynthesis’ and ‘ABC transporter’ (Additional file 20: Data S1), implying the importance of terpenoid regulation during flower development. As shown in Fig. 7a, we manually selected some candidate genes, including P, C, T, F, *MADS* and several terpenoid metabolism-related TFs, in each co-expression module. We noted that genes associated with ‘monoterpenoid biosynthesis’ were significantly enriched in M16 (Fig. 7b). Complex interaction was found among 25 and 13 genes for terpenoid biosynthesis and transport, 34 genes belonging to the CYP450s, 9 TFs and 29 genes related to flowering (Fig. 7c, Additional file 10: Figure S10). Thereinto, 23 terpenoid biosynthesis genes were predicted with ORF. Based on the phylogeny and functions of known TPSs, four subfamilies of TPSs of M16 are recognized, including 2 proteins for TPS-a, 11 for TPS-b, 7 for TPS-d and 3 for TPS-e/f (Fig. 8a). 34 CYP450 proteins of M16 were classified into four clans. Notably, there are 20 and 4 proteins dispersed into clans associated with terpenoid catalysis [15]——71 clan and 85 clan, respectively (Fig. 8b).

### Verification of gene expression

**Fig. 9:**
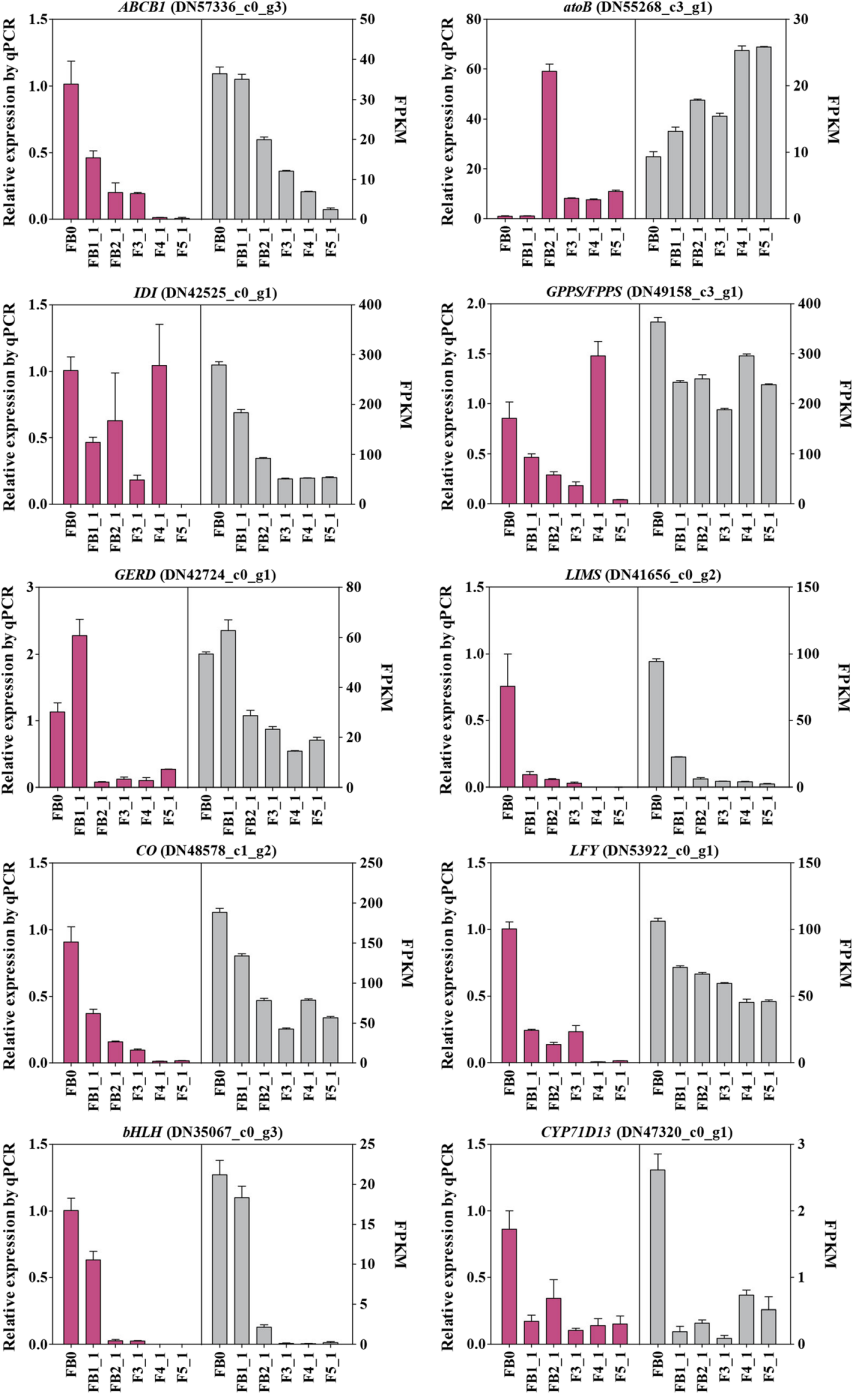
Expression patterns of eight genes as verified by qRT-PCR. Purple bars represent the relative expression levels of FB0 and first-axis flowers of five developmental stages, normalized to that of *actin* and *18S rRNA* transcripts. Grey bars indicate FPKM values from RNA-Seq. Values shown are mean ± SE of three replicates

To confirm the reliability of our transcriptome data, the expression levels of eight candidate transcripts involved in subnetwork of M16 were determined via quantitative real-time PCR (qRT-PCR). In our analysis, the expression patterns of total eight genes related to terpenoid biosynthesis (*atoB*, *GPPS/FPPS* and *LIMS*), modification (*CYP71D13*), transportation (*ABCB1*) and flowering (*bHLH*, *LFY* and *CO*) were generally in good agreement with the transcriptome data (Fig. 9; Additional file 11: Figure S11).

## Discussion

### Ecological implications of lavender inflorescences and emissions

The cross-pollination behaviour of lavender species fundamentally determines its dispersal by means of external forces, and insect pollinators play an important role in lavender reproduction [4]. The ringent two-lipped corolla, glandular trichome-rich calyx, protandry and multi-flower cincinnus of lavender elevated the ratio of intraspecific gene flow, giving rise to genetic diversity to enable adaption to hostile environments [3, 4]. Our results showed that inflorescences with sequential and successive blossoms leads to long flowering-life and lasting attraction to bees. The fine-tuning of the blossoming sequence of lavender may be ecologically significant for the repeated and persistent visitation of pollinators. Concurrently, numerous and diverse volatiles emitted by flowers act as long-distance signals and important cues for attracting pollinators to spread pollen, which is believed to be an evolutionary advantage that helps plants preserve offspring in unfavourable conditions [7, 16]. In this study, we identified a total of 56 mono- and sesquiterpenes in the EOs of *L. angustifolia* flower for the first time, and these terpenoids can be classified into four groups according to the maturity of individual florets (Fig. 2). Regardless of the sites of florets on the first or second axis, the changes in qualitative and quantitative terpenoids were closely related to flower development, indicating that the maturity levels determine the changes in compounds rather than the sites of florets.

Simultaneously, phytochemical diversity drives plant–insect community diversity [17, 18]. Lavender-visiting insects, including herbivores, natural enemies and pollinators, were investigated for the first time along with the maturity of lavender inflorescence. Mounting evidence has highlighted the role of volatile terpenoids in communication between plants and insects [16, 17, 19]. The flower buds of lavender emerge in early April at north of China, when the weather is dry and rainless and aphids multiply quickly. Besides lavender, many other plants, such as mint (*Mentha* × *piperita*), peach tree *(Amygdalus persica*), pepper (*Zanthoxylum bungeanum*) and *Hemistepta lyrata*, were attacked heavily during this period of time. Our study showed that (+)-R-limonene and *β*-trans-ocimene are the two highest compounds in the EOs of FB0, and can get rid of aphids in Y-tube bioassay. Previous studies have reported that monoterpenes such as limonene, linalool or ocimene, are usually released within 24 h after attack and plants overexpressed genes encoding linalool, limonene, cymene and ocimene-related synthetases exhibit higher activity in inhibiting infestation of aphids [20–24]. Transcriptomic dynamics over various developmental stages revealed that *LIMS* and *BOS* expressed strongly in tender and succulent FB0 sample, which is one of prospective host of aphids. Therefore, we supposed that the original bud of FB0 could release some specific volatiles (limonene, *β*-ocimene and *α*-pinene etc.) to defend aphids, resulting that the lavender can prevent further damage caused by herbivores and get through this key developmental stage. In addition to the direct defence by plants themselves, predatory insects attracted by lavender such as ladybirds, lacewings, spiders, and hoverflies may contribute to eliminating the aphids. Many reports have demonstrated that an array of volatiles, referred to as herbivore-induced plant volatiles, are produced when plants encounter aggressive insects, resulting in the attraction of natural enemies [22, 23]. Taken together, the development of lavender inflorescences and associated volatiles may play an important role in structuring plant-associated insect communities to accommodate survival and the evolutionary ‘arms race’.

The most easily and frequently observed events were the initial visits by honeybees and other pollinators when the first floret opened. Three typical compounds, linalool, linalyl acetate and lavandulyl acetate, accounted between 69.17 to 81.49% of the total lavender EOs except in FB0 (19.44%), implying their crucial roles in responses to biotic and abiotic stress or mediating interactions between plants and visitors. Interestingly, the contents of linalool/linalyl acetate and linalool/lavandulyl acetate showed opposite trends with flower development; for example, decreased linalool and elevated linalyl acetate and lavandulyl acetate were found at anthesis (Fig. 2a). It has been reported that lavandulol could be converted into lavandulyl acetate by the acetyltransferase enzyme (AAT3 and AAT4) in vitro, and an unknown acetyltransferase enzyme may be involved in the conversion of linalool and linalyl acetate [24]. Hence, more efforts need to focus on deciphering the synthesis of terpenoids and their derivatives at the genetic level and further resolving the relationship between these compounds. Additionally, a laboratory experiment found that linalyl acetate and lavandulyl acetate enhanced attractiveness to bees more effectively than linalool, possibly due to the pleasant aroma of monoterpene esters [2]. In contrast to a previous study that documented linalool as an attractant for pollinators [25], the linalool content decreased at anthesis but increased during the fading stage in our study, suggesting that linalool may act as a repellent to protect lavender seeds against damage from insects.

### Network analysis reflects the correlation between flowering time and terpenoid metabolism at the molecular level

The flow of emissions of indeterminate thyrse inflorescence in lavender ensured the continual visitation of pollinators and plant survival through history. Blooming, accompanied by changes in volatiles, is one of the major factors affecting the interaction between lavender and insects. However, the relationship between the floral scent and flowering time is rarely examined at molecular level. A distinct terpenoid profile was detected in FB0 compared to other periods in our study, suggesting that this stage may initiate a process of readjustment along with the activation of key regulators in the development of first- and second-axis flowers [26]. Likewise, microarray data on FFDSFSA compared to FB0 revealed a dazzling diversity of gene expression. We identified abundant DEGs related to flowering time and terpenoid metabolism, providing rich information on the regulators of volatiles.

Our network analysis confirmed the superiority of WGCNA in analysing highly multivariate and complex data. A detailed and clear insight into the spatiotemporal dynamics associated with the maturity of lavender flower has been obtained via WGCNA. The temporal regulation of gene expression plays an important role in plant growth and development. Detailed information about gene expression is crucial for understanding the molecular mechanisms underlying any developmental process. Recently, integrated analysis of gene co-expression and terpenoid accumulation has provided new insights into the regulatory processes of terpenoid metabolism [8]. CYP450s, which typically catalyse irreversible reactions and consequentially represent ideal points of control for metabolic bifurcation, are the major source of the chemical diversification of terpenoids, especially members of the CYP71 clan. In our work, many CYP450s, such as *CYP71D13*, *CYP71A1*, and *CYP76AH1*, were co-expressed with genes related to terpenoid biosynthesis. Increasing evidence indicates that many TPS/CYP450 gene pairs are found together in multiple sequenced plant genomes [15]. Gene–metabolite co-expression analysis has successfully identified novel *CYP450* genes involved in terpenoid indole alkaloid biosynthesis and regulation in periwinkle (*Catharanthus roseus*) [27] and two *CYP450* genes involved in triterpene saponin glycyrrhizin biosynthesis in Glycyrrhiza plants [28]. In Arabidopsis, *CYP71B31* and *CYP76C3*, together with *TPS10* [form (2)-(R)-linalool] and TPS14 [form (+)-(S)-linalool], are co-expressed in flowers at anthesis and catalyse the oxidation of the two enantiomers of linalool to produce a sets of hydroxylated or epoxidized products [29]. *CYP76C1*, which was co-expressed with *TPS10* and *TPS14*, was also identified as a major linalool metabolizing oxygenase in Arabidopsis [25]. These subsequent oxidative steps are catalysed by members of the CYP450 superfamily, indicating the coordination of TPS and CYP450s in producing impressively diverse terpenoids that play important roles in the ecological interaction of plants with biotic and abiotic stress.

Numerous reports have illustrated that delayed flowering can increase the accumulation of primary metabolism products in plants [30], while few effects of alteration in the flowering time on secondary metabolism are known. Lv et al. (2018) demonstrated that inhibition of *AaFT2* may delay the flowering time and increase the accumulation of artemisinin in transgenic *A. annua*, but it is not clear that how flowering time impacted the contents of terpenoids [31]. In *Petunia hybrida* and *N. attenuate*, reducing the expression of the clock gene, *LATE ELONGATED HYPOCOTYL* (*LHY*), advanced the phase of scent emission as well as expression of genes in the floral volatile benzenoid/phenylpropanoid pathway [32, 33]. The oscillation of monoterpenoid and sesquiterpenoid emission is a consequence of the regulation of the gene encoding key enzymes in MEP and MVA pathway, which is also possibly controlled by the circadian clock. However, the interaction mechanism between flowering time-related genes and terpenoid metabolism-related genes in aromatic plants remained elusive. Our findings suggest a regulatory network involved in both in flowering time and volatile terpenoid and the potential link between them. As our current understanding of the transcriptional regulation of the flowering time and MEP/MVA pathway is still incomplete, a further investigation into the explicit and specific relationship is pending.

The members of the *WRKY*, *MYB* and *bHLH* families and the well-known *MADS*, which showed co-expression with terpenoid metabolism-related genes, have been reported to orchestrate flower development in many plant species [34]. Evidence from *Arabidopsis* has demonstrated that *WRKY75* positively regulates flowering in an FLT-dependent manner, but *RGL1* and *GAI* can repress the activation ability of *WRKY75*, thereby partially rescuing the early flowering phenotype of *WRKY75*-overexpressing plants [35]. Several TFs appeared to be extensively correlated with most terpenoid metabolism-related genes in the subnetwork, implying that they may regulate an upstream step in plant secondary metabolism. It has already been proven that *MsMYB* is a negative regulator of monoterpene biosynthesis in spearmint [36]; *MsYABBY5* RNAi lines exhibit a 20%~ 77% increase in monoterpene production [37]; *SlWRKY73* can transactivate the *SlTPS5* promoter in tomato [38]; and *AaWRKY1* regulates the amorpha-4,11-diene synthase gene, which catalyses a committed step of artemisinin biosynthesis [39]. In conclusion, the above candidates opened the door for trailblazing discoveries of the molecular underpinnings of terpenoid regulation. The expression or modulation of those functional homologs in lavender might thus be considered for applications in improving EO quality and biological control.

## Conclusions

This study provides, to our knowledge, the first comprehensive view of changes in gene expression and volatiles during the flower developmental reprogramming of lavender. We successfully profiled the transcriptome and volatiles of lavender flowers and detected detailed changes at both the transcriptomic and metabolic levels via an omics plus bioinformatics approach. Characteristic compounds and gene expression profile of FB0 exhibit ecological value in pest control. The precise control of each-axis flowering and regular emissions at transcriptional and metabolic level are important to pollinators attraction for lavender. Our study sheds new light on the ecological and genetic stability and flexibility of lavender inflorescences from “gene-volatile terpenoid-insect” three layers.

## Methods

### Plant material collection

The experiment was carried out at the experimental farm, Aromatic Plants Resources Development and Engineering Laboratory of Xinjiang production and Construction Corps, Yili, Xinjiang (43°50′9.66″N, 81°10′21.73″E), during spring-summer (from 10th, May to 29th, June) season of 2016. Samples were collected from 2-year-old *L. angustifolia* ‘JX-2’ bred by our laboratory, which is planted in row width of 1 m apart, with 50 cm between plants. The voucher specimen of *L. angustifolia* ‘JX-2’ was kept at the Chinese national herbarium, Institute of Botany, Chinese academy of sciences (voucher specimen: 02308796). To maintain the uniformity of plant material, the first and second axes of florets from the remote whorl of *L. angustifolia* ‘JX-2’, which clusters five discontinuous whorl florets in a spike, were selected for in-depth study. The two symmetric adaxial florets (indicated by ‘1’ in Fig. 1) opened first from the base to the terminal of one rachis, followed by the second-axis florets (indicated by ‘2’ in Fig. 1) developed from each first axis. To distinguish the two-axis florets better, we named the florets “stage_axis”: here, “stage” referred to the flower developmental stages defined in Guitton et al. [5], where “F” means flower, “FB” means flower bud and “1–5” five degrees of maturity, and the stages of the second-axis flowers were named in the same pattern as the first-axis flowers.

In total, we collected 33 flower samples, including an original bud as well as the two-axis florets of five maturity stages in a remote whorl, each with three replicates. Harvested flowers were divided into two portions: the first was immediately frozen in liquid nitrogen and stored at − 80 °C for RNA extraction, and the other was placed in the shade to dry for chemical component identification.

### Essential oil extraction and GC-MS analysis

A total of 20 g of dry lavender florets at different developmental stages were collected, and the essential oil of the lavender was extracted by water distillation followed by soaking in distilled water for 1 h. All of the isolated essential oil samples were dried over anhydrous sodium sulphate and stored at 4 °C prior to analysis by GC-MS. GC-MS analysis was performed on an Agilent 7890A GC system and an Agilent Technologies 5975C Inert XL Mass Selective Detector, equipped with an HP-5MS UI column (30 m × 0.25 mm × 0.25 μm; Agilent Technologies).

The conditions were as follows: samples were diluted in hexane at a ratio of 1:100, and 0.8 μl of sample was injected in split mode (1:20). The injector temperature was 250 °C, and the oven program was as follows: 40 °C for 2 min, linear ramp at a rate of 4 °C·min^− 1^ to 260 °C, second ramp to 310 °C at 60 °C·min^− 1^, hold at 310 °C for 15 min. The transfer line temperature was 280 °C. Helium was used as the carrier gas at a flow rate of 1.0 mL·min^− 1^ through the column. The MS conditions were as follows: ionization energy, 70 eV; electronic impact ion source temperature, 200 °C; quadrupole temperature, 150 °C; and mass range, 40–600 u.

Agilent MassHunter 5.0 was used to analyse the chromatograms and mass spectra. The constituents of the essential oils were identified by comparing the retention times of individual peaks with the retention times of the reference and by identifying the mass spectra using the mass spectra databases NIST 2014 and literature data [2].

### RNA isolation, library construction and sequencing

Total RNA was extracted from 33 finely ground flower samples using a HiPure Plant RNA Mini Kit (Magen) according to the manufacturer’s instructions. The DNA was digested by DNaseI (Magen). RNA purity, concentration and integrity were determined using Nanodrop 1000 spectrophotometer, Qubit Flurometer and Agilent Bioanalyzer. Only the RNA samples with 260/280 ratio between 1.8 to 2.1, 260/230 ratio between 2.0 to 2.5 and RIN (RNA integrity number) more than 8.0, were used for sequencing. Qualified RNA was enriched with oligo (dT)-rich magnetic beads and then broken into short fragments in Fragmentation Buffer. 1st strand cDNA synthesis was performed using random hexamers primer and M-MuLV Reverse Transcriptase (RNase H). 2nd strand cDNA was synthesized by adding reaction buffer, dNTPs, RNase H and DNA polymerase I. Next, the resulting cDNAs were subjected to end-repair, insert ‘A’ base and subsequently ligate with Illumina paired end solexa adaptor. Adaptor-ligated fragments were purified by AMPure XP beads to select a size range of templates for downstream enrichment. Finally, PCR was performed to enrich and amplify the cDNA template. In total of 33 libraries including three biological replicates for each sample were constructed and then sequenced on Illumina HiSeq™ 2000 platform at Novogene Biotechnology Corporation (Beijing, China). We obtained a total of 1737,289,686 (1737 million) clean reads and at least 6.25 Gb clean data per library was generated after filtering and removing the adapter sequences from the raw data (Additional file 14: Table S3). The value of Q20, Q30 and GC content was higher than 96.06, 91.54 and 46.86%, respectively. When we mapped the reads back to the assembled unigenes, the mapping rate ranged from 77.40 to 80.03% of each library (Additional file 14: Table S3). RNA sequencing raw sequence data of 33 lavender flower samples generated from the present study can be found in the National Center for Biotechnology Information (NCBI) Short Read Archive database with accession number SRP139393.

### De novo transcriptome assembly and unigene annotation

De novo assembly of the processed reads was carried out using the Trinity program (r20140413p1) with min_kmer_cov set to 2 by default and all other parameters set to default. For functional annotation, unigenes were used as query sequences to search seven annotation databases. Using NCBI blast (v2.2.28+), the unigenes were annotated to NR, NT, Swiss-Pro (*e*-value = 1e-5), KOG/COG (*e*-value = 1e-3) databases. For KEGG, Pfam and GO annotation, KAAS (r140224), hmmscan (HMMER 3) and blast2go (b2g4pipe_v2.5) were used with thresholds of 1^e-10^, 0.01 and 1^e-6^, respectively. Moreover, GO enrichment analysis was implemented by using the topGO R packages based on the Kolmogorov–Smirnov test (*P* < 0.05). We used KOBAS software (v2.0.12) to test the statistical enrichment of genes (*P* < 0.05) in the KEGG pathways.

### DEG analysis

The expression abundance of corresponding unigenes was represented by fragments per kilobase of transcript sequence per millions of base pairs sequenced (FPKM). The DEGs between various samples were identified and filtered with the R package DESeq. We used FDR < 0.01 and the absolute value of log_2_(ratio) ≥ 2 as thresholds to define differential gene expression. The FPKM between the biological replications was analysed using Pearson’s correlation coefficient (r) and the closer the r^2^ value to 1, the stronger was the correlation between samples. The highest r value among three biological replicates samples of each developmental stage indicated the stability and reproducibility of the data (Additional file 4: Figure S4).

### Gene co-expression network construction and visualization

A co-expression gene network was constructed using the WGCNA software package (v1.51) in R using all DEGs. Modules with default settings, except that the power is 8, minModuleSize is 20, and minimum height for merging modules is 0.33805. Genes with the highest degree of connectivity within a module are referred to as intramodular hub genes [14]. The networks about hub-genes were visualized using Cytoscape (v.3.0.0).

### Insect survey in the field and olfaction bioassays in the laboratory

Flower visits of insects were observed from 10:00 to 11:00 am in the field from bud appearing to blossom ending. The number of visitors was recorded during different flower developmental stages. For laboratory experiments, the behavioural responses to specific mono- or sesquiterpenoids of *A. mellifera* (at uniform growth and activity) and *Rhopalosiphum padi* were conducted in a Y-tube olfactometer at 22 ± 2 °C (room temperature). Two glass vessels containing an odour source (2 μl of white oil or a standard substance diluted to a specific percentage: 20.57% (v/v) of *β*-trans-ocimene, 17.0% (v/v) of (+)-(R)-limonene, which is similar to the concentration ratio of FB0; 32.2% of linalyl acetate, 16.3% of lavandulyl acetate, 30.8% of linalool (which is similar to the concentration ratio of F3_1) were connected to the arms of the Y-tube olfactometer using Teflon tubes. A fluorescent light at an intensity of 30–35 μmol photons m^− 2^ s^− 1^ was used to illuminate the middle of the crotch of the Y-tube. The experiment started with the release of bees or aphids at the base of the Y-tube with ventilation. Each insect was observed for a maximum of 5 min, and a choice was recorded when the bee reached the middle of either arm and remained in that arm for at least 10 s. When the insect did not make a choice within 5 min, a ‘no choice’ behavioral response was recorded. Each experiment was repeated 3 times (i.e., 3 × 30 starved insects) for a particular concentration of odor source on 3 different experimental days with new groups of insects and new odor source per day. Experiments with other concentrations of odor sources were conducted in the same manner.

### qRT-PCR

The same RNA samples of microarray experiments were used for qRT-PCR. First-strand cDNA was synthesized with oligo (dT)_18_ and M-MLV reverse transcriptase (Promega). qRT-PCR analysis was carried out using the SYBR Fast Universal qPCR Kit (TSINGKE) on an Mx3000P system (Agilent Stratagene), according to the manufacturer’s instructions. The following standard thermal profile was used for all PCRs: predenaturation at 95 °C for 3 min and 40 cycles of denaturation at 95 °C for 15 s, annealing at 60 °C for 15 s, and elongation at 72 °C for 20 s. Quantification was performed using the 2^−ΔΔCT^ method, and data were normalized to those of the *18S rRNA* and *actin* transcript. Sequences of primers used are listed in Additional file 18: Table S7.

## Additional files

Additional file 1: **Figure S1.** GC–MS total ion chromatograms of volatiles collected from EOs of lavender flowers at different developmental stages. (JPG 191 kb)

Additional file 2: **Figure S2.** Heatmap of 56 compounds contents in 11 samples after normalized by z-scores. (JPG 358 kb)

Additional file 3: **Figure S3** Length distribution and annotation of Illumina assembled unigenes in lavender. (JPG 351 kb)

Additional file 4: **Figure S4** Density profile and box plot of FPKM of flower at different developmental stages and the correlation of expression level among 33 flower sample. (JPG 564 kb)

Additional file 5: **Figure S5.** Volcano Plots of DEGs between FFDSFSA and FB0. (JPG 194 kb)

Additional file 6: **Figure S6.** Venn diagrams of DEGs. (JPG 207 kb)

Additional file 7: **Figure S7.** Counts of differentially expressed TFs. (JPG 98 kb)

Additional file 8: **Figure S8.** Heatmap of genes involved in CYP450 family and terpenoid transport. (JPG 152 kb)

Additional file 9: **Figure S9.** Module-module and module-sample correlations. (JPG 159 kb)

Additional file 10: **Figure S10.** Details of subnetwork of M16. (JPG 392 kb)

Additional file 11: **Figure S11.** Expression patterns of eight genes during different developmental stages of second-axis flower as verified by qRT-PCR. (JPG 259 kb)

Additional file 12: **Table S1.** Previously cloned genes for biosynthesis of mono- and sesquiterpenoids in lavenders. (DOCX 13 kb)

Additional file 13: **Table S2.** Contents of major mono- and sesquiterpenoids identified in EOs of lavender. (DOCX 86 kb)

Additional file 14: **Table S3.** Information of Illumina reads from 33 libraries. (DOCX 16 kb)

Additional file 15: **Table S4.** Annotation of all unigenes based on seven databases. (DOCX 13 kb)

Additional file 16: **Table S5.** Summary of putative genes of CYP450s family in lavender. (DOCX 15 kb)

Additional file 17: **Table S6.** Summary of putative terpenoid transporters in lavender. (DOCX 12 kb)

Additional file 18: **Table S7.** Gene-specific primer pairs used for qRT-PCR. (DOCX 13 kb)

Additional file 19: **Video S1.** Animation schematically illustrates the sequence of blossom in lavender. (PPTX 5149 kb)

Additional file 20: **Data S1.** Summary of top 20 KEGG enrichment pathways of 21 modules produced by WGCNA. (XLSX 30 kb)

## Data Availability

All data generated or analysed during this study are included in this published article and its supplementary information files.

## Abbreviations

AAT: Acetyltransferase enzyme
AP1: Apetala 1
C: Cytochrome P450 hydroxylases
CO: Constans
CYP450s: Cytochrome P450 monooxidase
DEGs: Differentially expressed genes
DXS: Deoxy-D-xylulose-5-phosphate synthase
EO: Essential oil
FDR: False discovery rate
FFDSFSA: Flowers of five developmental stages at first- and second-axis
FLT: Flowering locus T
FPKM: Fragments per kilobase of transcript sequence per millions base pairs sequenced
GRED: (−)-germacrene D synthase
HDR: 4-hydroxy-3-methylbut-2-en-1-yl diphosphate reductase
HDS: (E)-4-hydroxy-3-methylbut-2-enyl-diphosphate synthase
IDI: Isopentenyl-diphosphate delta isomerase
LFY: Leafy
LIMS: Limonene synthase
LINS: Linalool synthase
ME: Module eigengene
MVD: Mevalonate diphosphate decarboxylase
P: Terpenoid synthesis pathway
SabS1: Sabinene synthase
SOC1: Suppressor of overexpression of constans 1
T: Terpenoid transporters
TEPS: α-terpineol synthase
TFs: Transcription factors
TPS: Terpene synthase
WGCNA: Weighted gene co-expression network analysis
