# Supplementary figures and images for: Time-series transcriptome provides insights into the gene regulation network involved in the volatile terpenoid metabolism during the flower development of lavender
Source: BMC Plant Biol. 2019 Jul 15;19:313. doi: 10.1186/s12870-019-1908-6 (PMC6632208; doi:10.1186/s12870-019-1908-6)

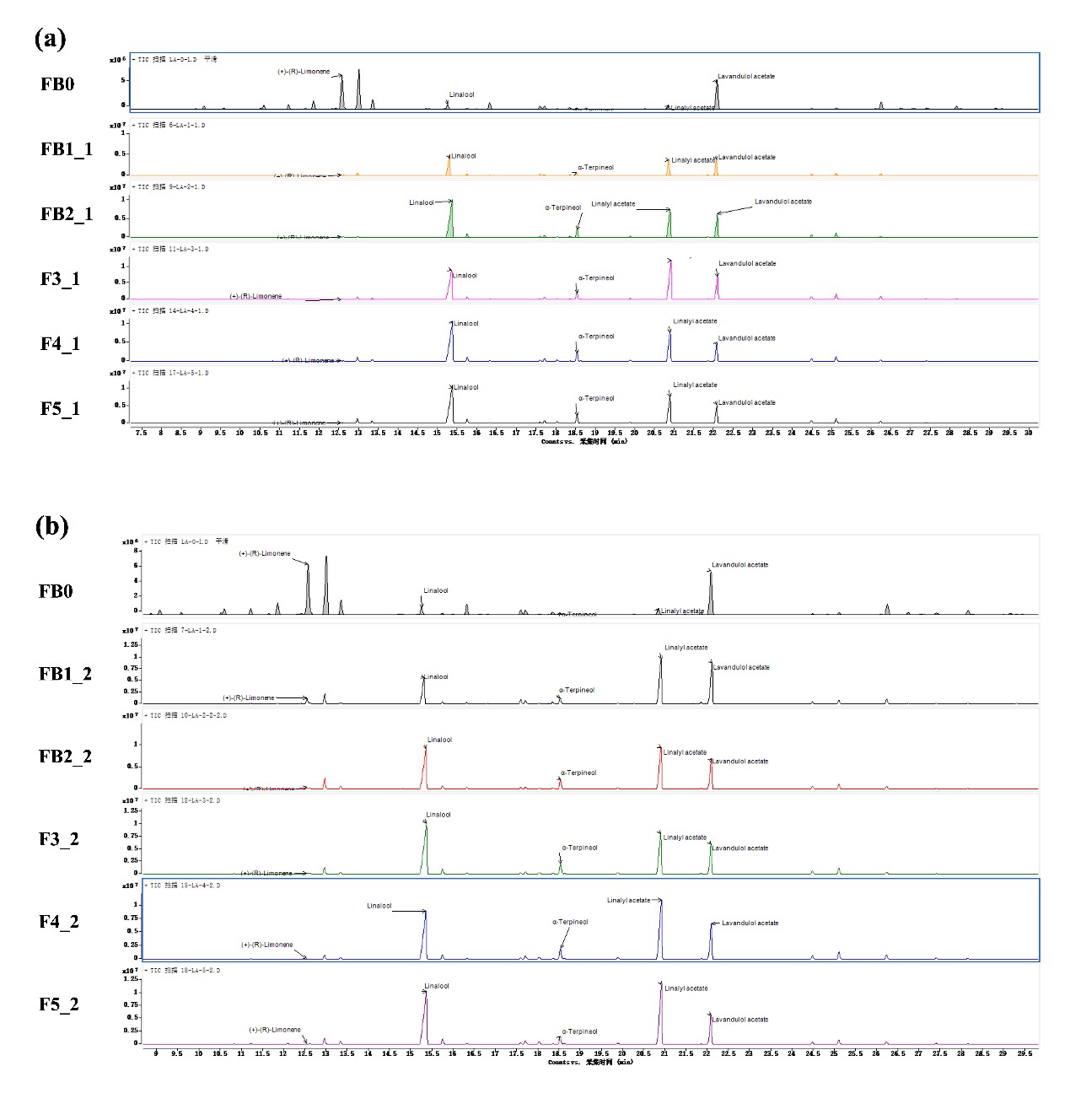

Supplement: Supplementary file 1 — Figure S1. GC–MS total ion chromatograms of volatiles collected from EOs of lavender flowers at different developmental stages. (JPG 191 kb) [file 12870_2019_1908_MOESM1_ESM.jpg]

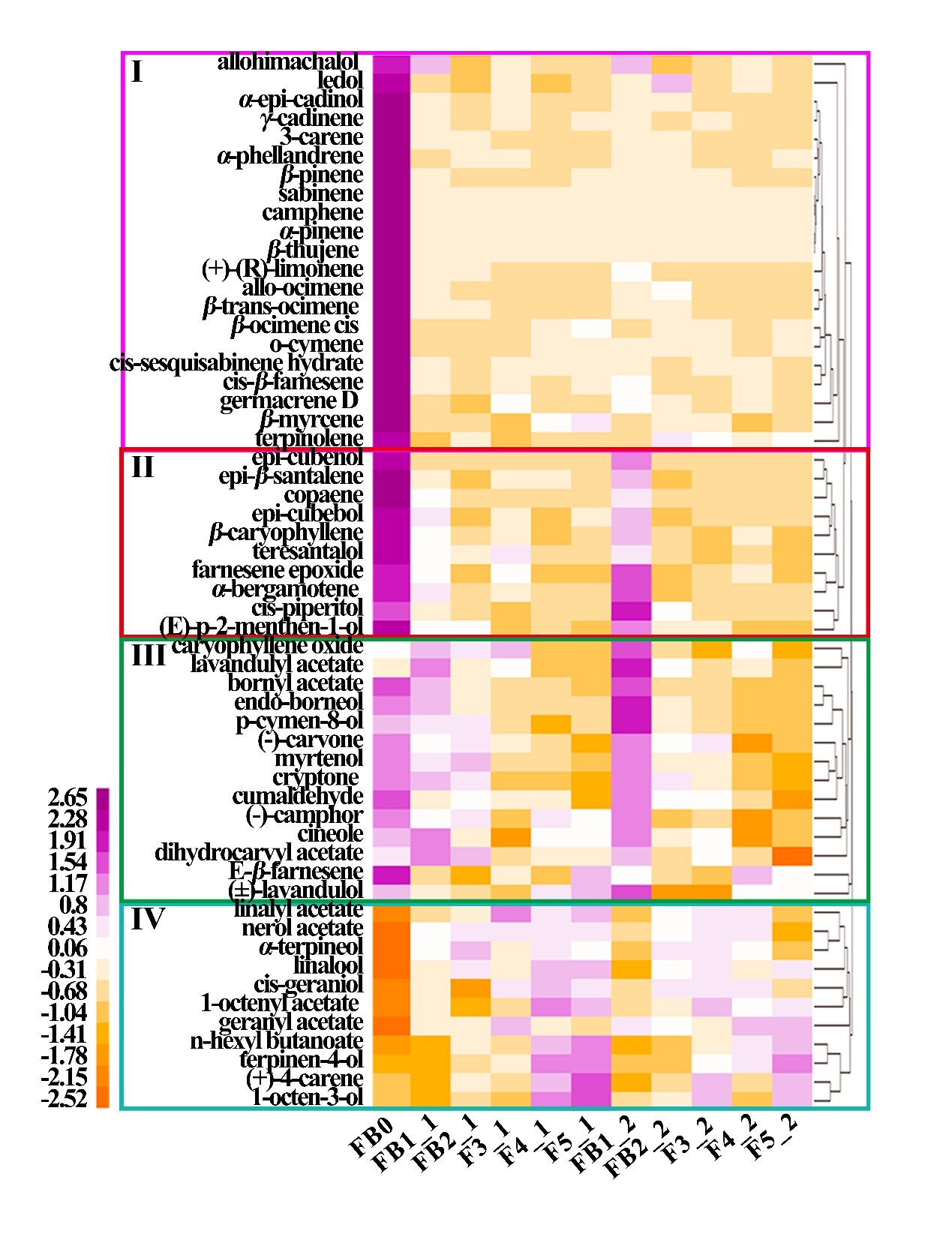

Supplement: Supplementary file 2 — Figure S2. Heatmap of 56 compounds contents in 11 samples after normalized by z-scores. (JPG 358 kb) [file 12870_2019_1908_MOESM2_ESM.jpg]

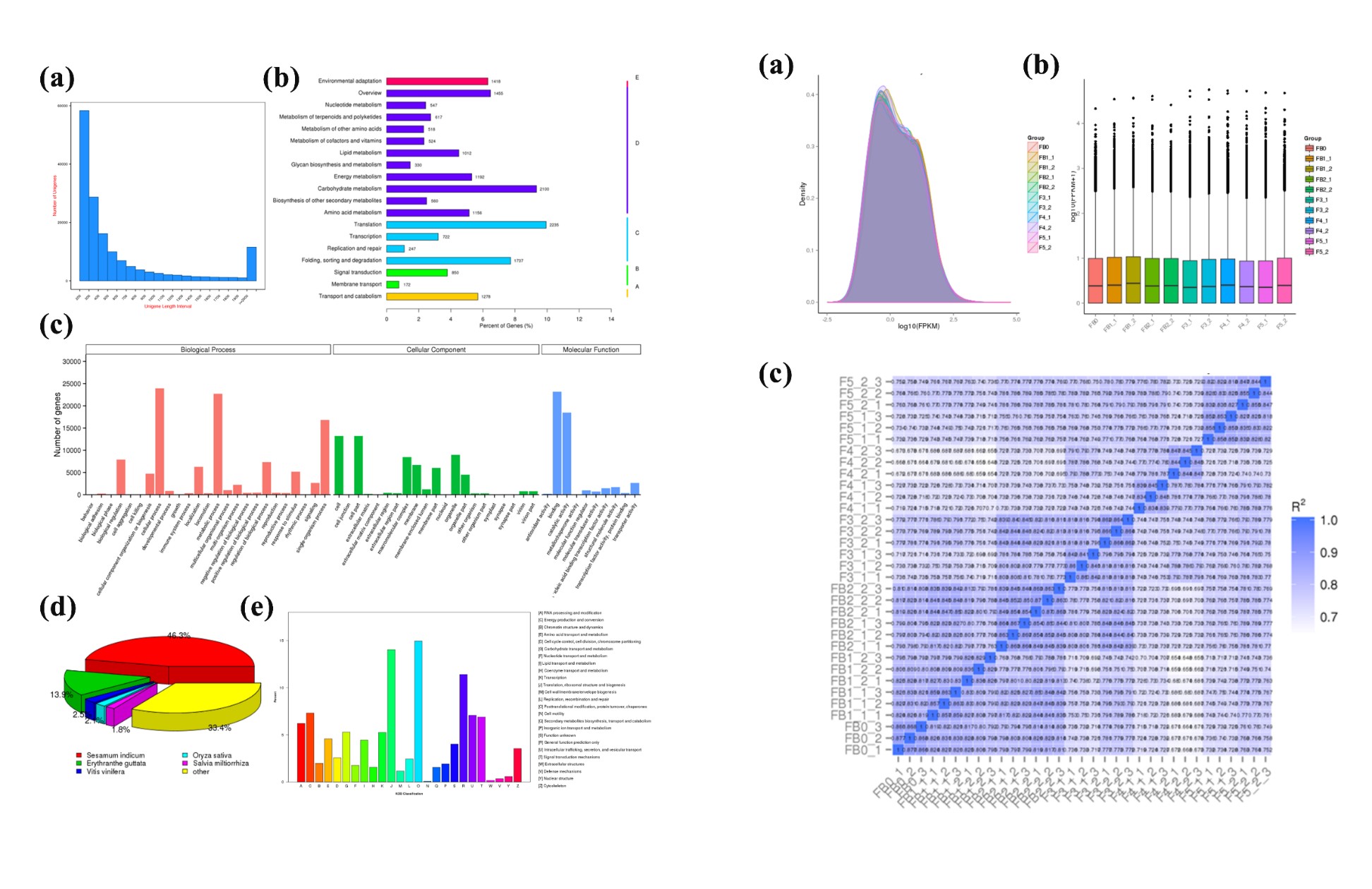

Supplement: Supplementary file 3 — Figure S3 Length distribution and annotation of Illumina assembled unigenes in lavender. (JPG 351 kb) [file 12870_2019_1908_MOESM3_ESM.jpg]

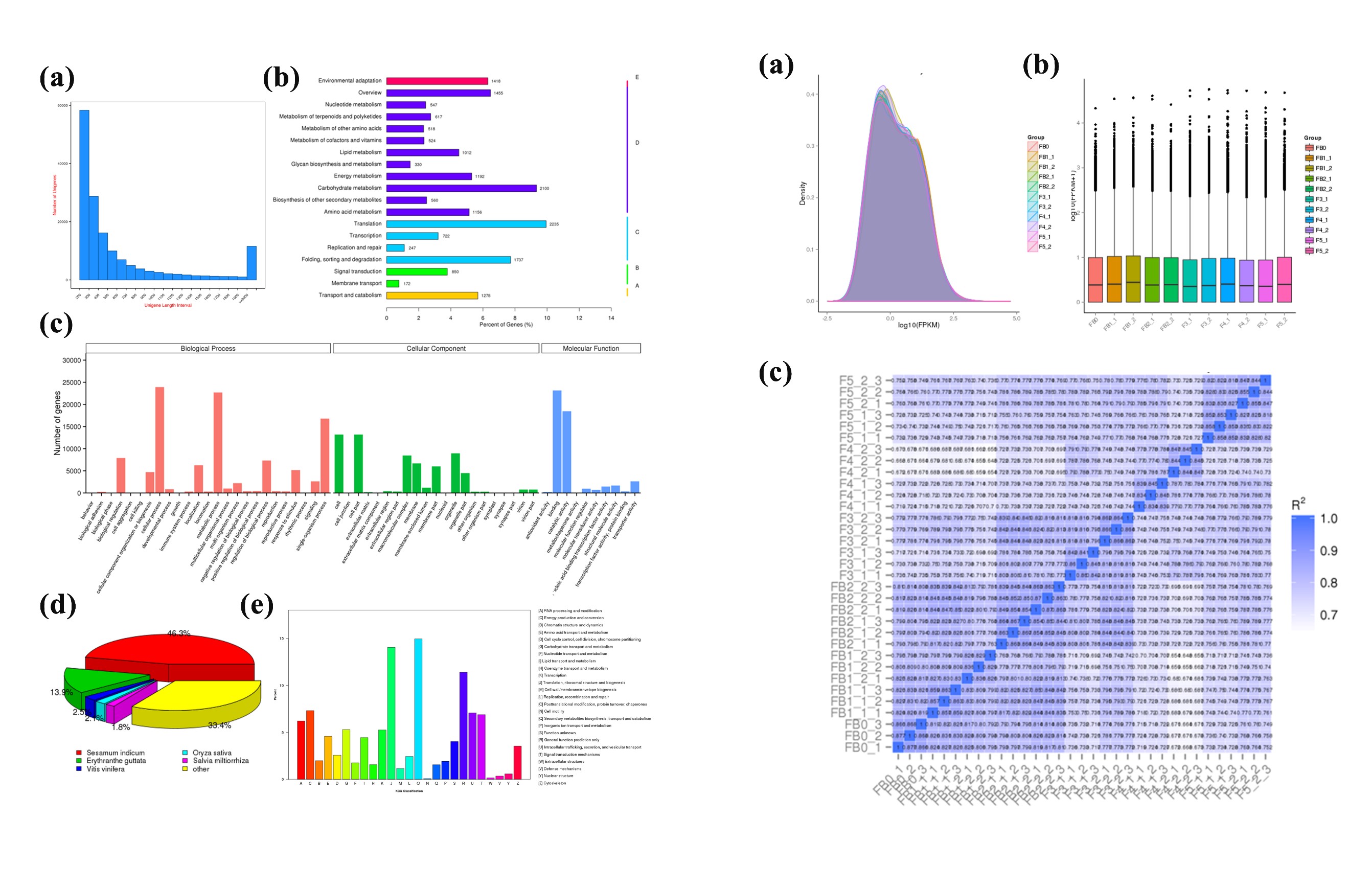

Supplement: Supplementary file 4 — Figure S4 Density profile and box plot of FPKM of flower at different developmental stages and the correlation of expression level among 33 flower sample. (JPG 564 kb) [file 12870_2019_1908_MOESM4_ESM.jpg]

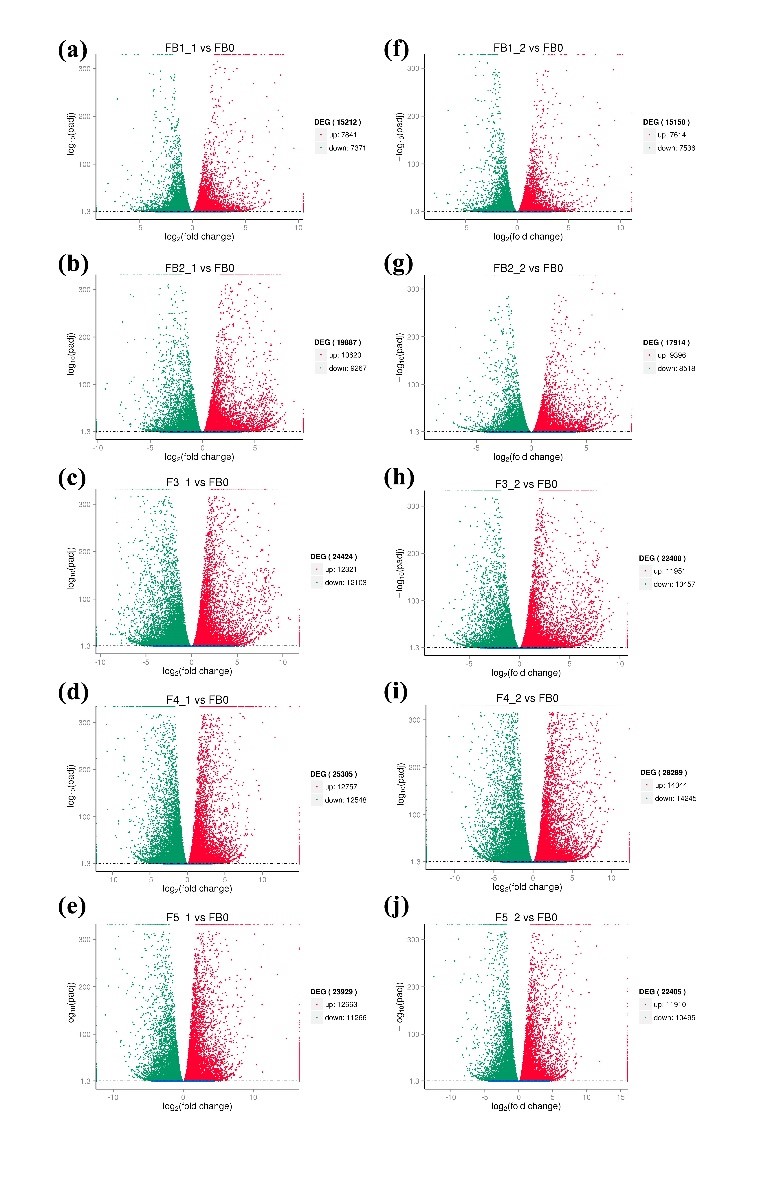

Supplement: Supplementary file 5 — Figure S5. Volcano Plots of DEGs between FFDSFSA and FB0. (JPG 194 kb) [file 12870_2019_1908_MOESM5_ESM.jpg]

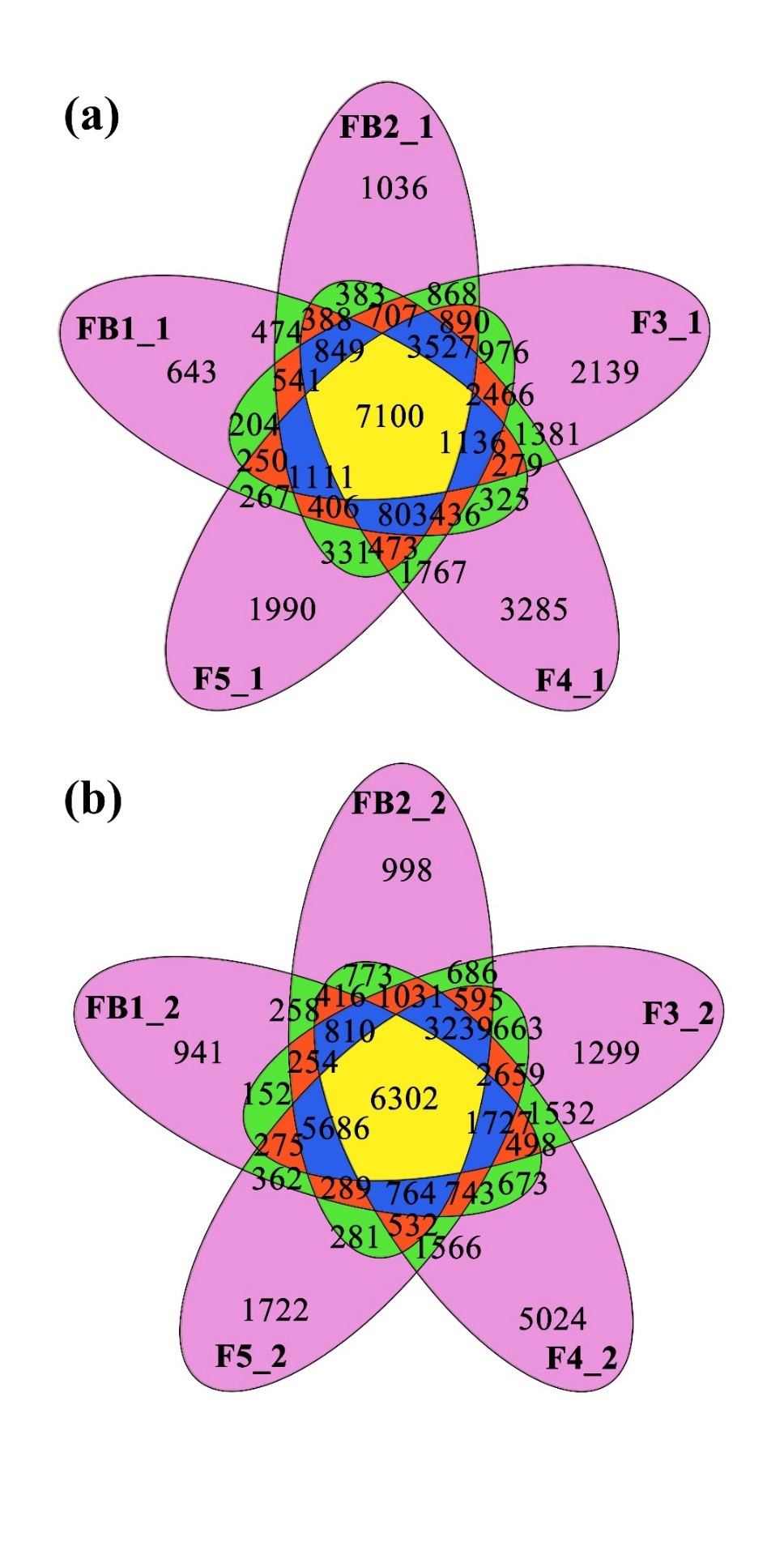

Supplement: Supplementary file 6 — Figure S6. Venn diagrams of DEGs. (JPG 207 kb) [file 12870_2019_1908_MOESM6_ESM.jpg]

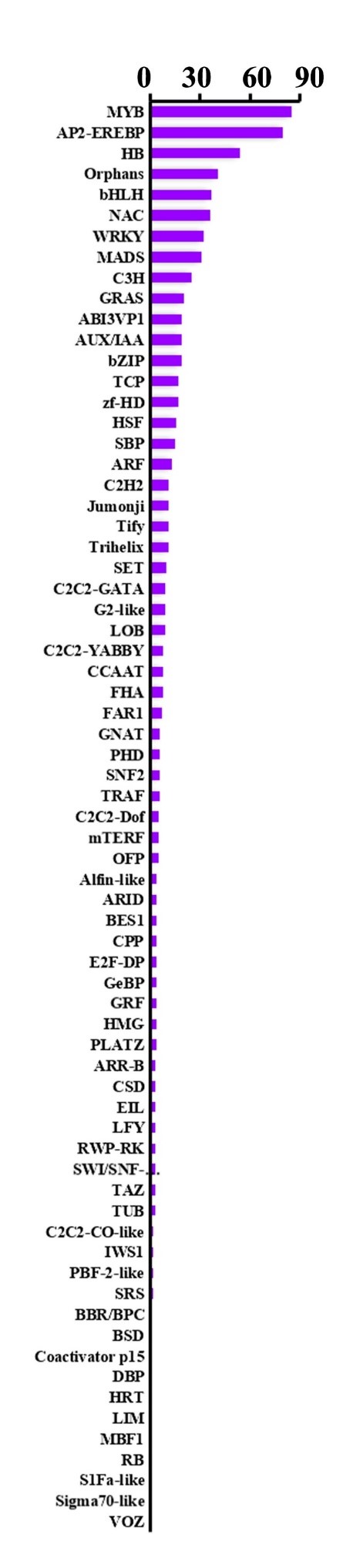

Supplement: Supplementary file 7 — Figure S7. Counts of differentially expressed TFs. (JPG 98 kb) [file 12870_2019_1908_MOESM7_ESM.jpg]

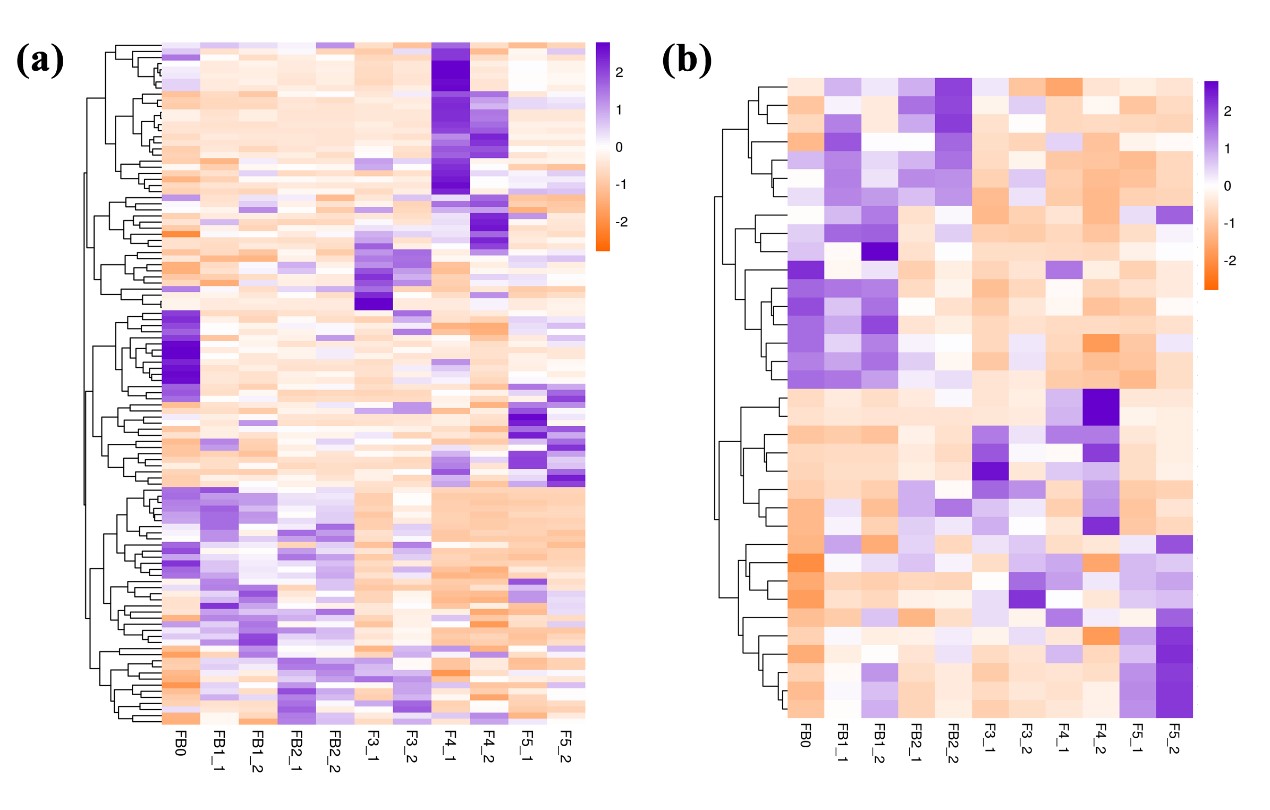

Supplement: Supplementary file 8 — Figure S8. Heatmap of genes involved in CYP450 family and terpenoid transport. (JPG 152 kb) [file 12870_2019_1908_MOESM8_ESM.jpg]

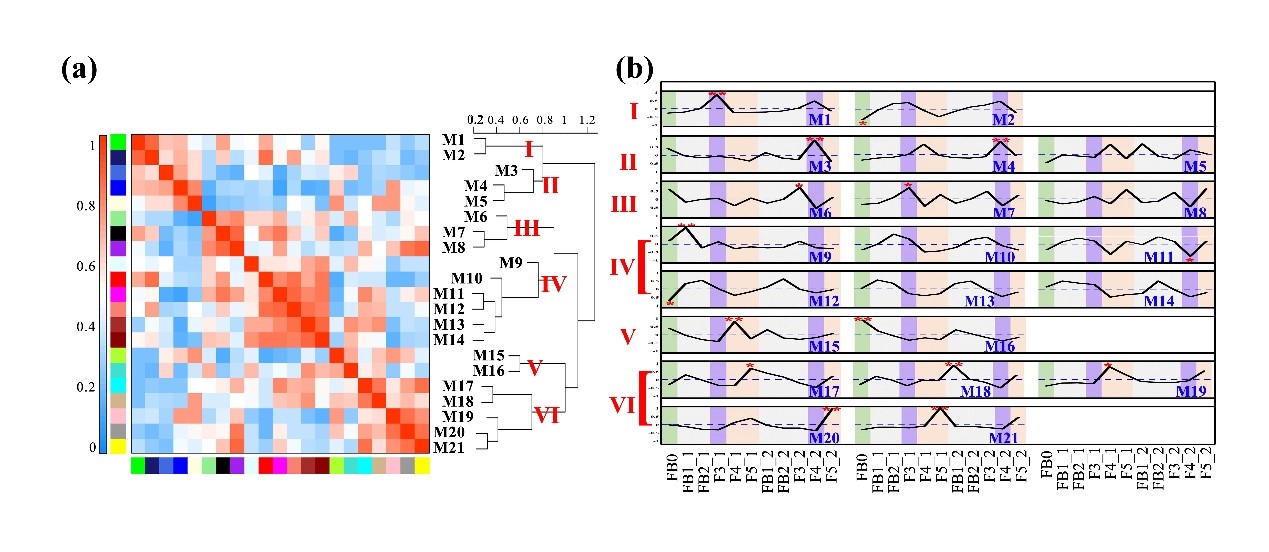

Supplement: Supplementary file 9 — Figure S9. Module-module and module-sample correlations. (JPG 159 kb) [file 12870_2019_1908_MOESM9_ESM.jpg]

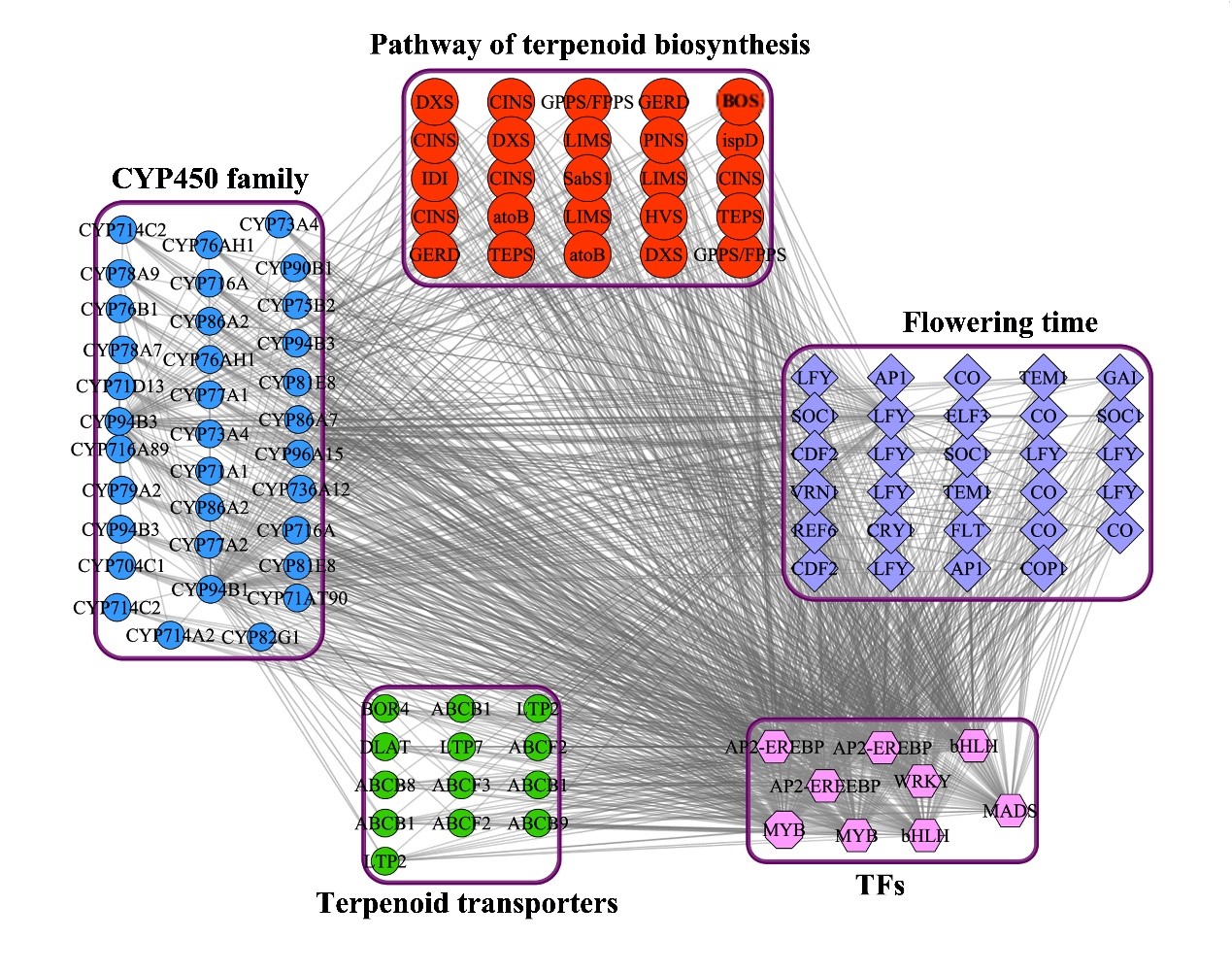

Supplement: Supplementary file 10 — Figure S10. Details of subnetwork of M16. (JPG 392 kb) [file 12870_2019_1908_MOESM10_ESM.jpg]

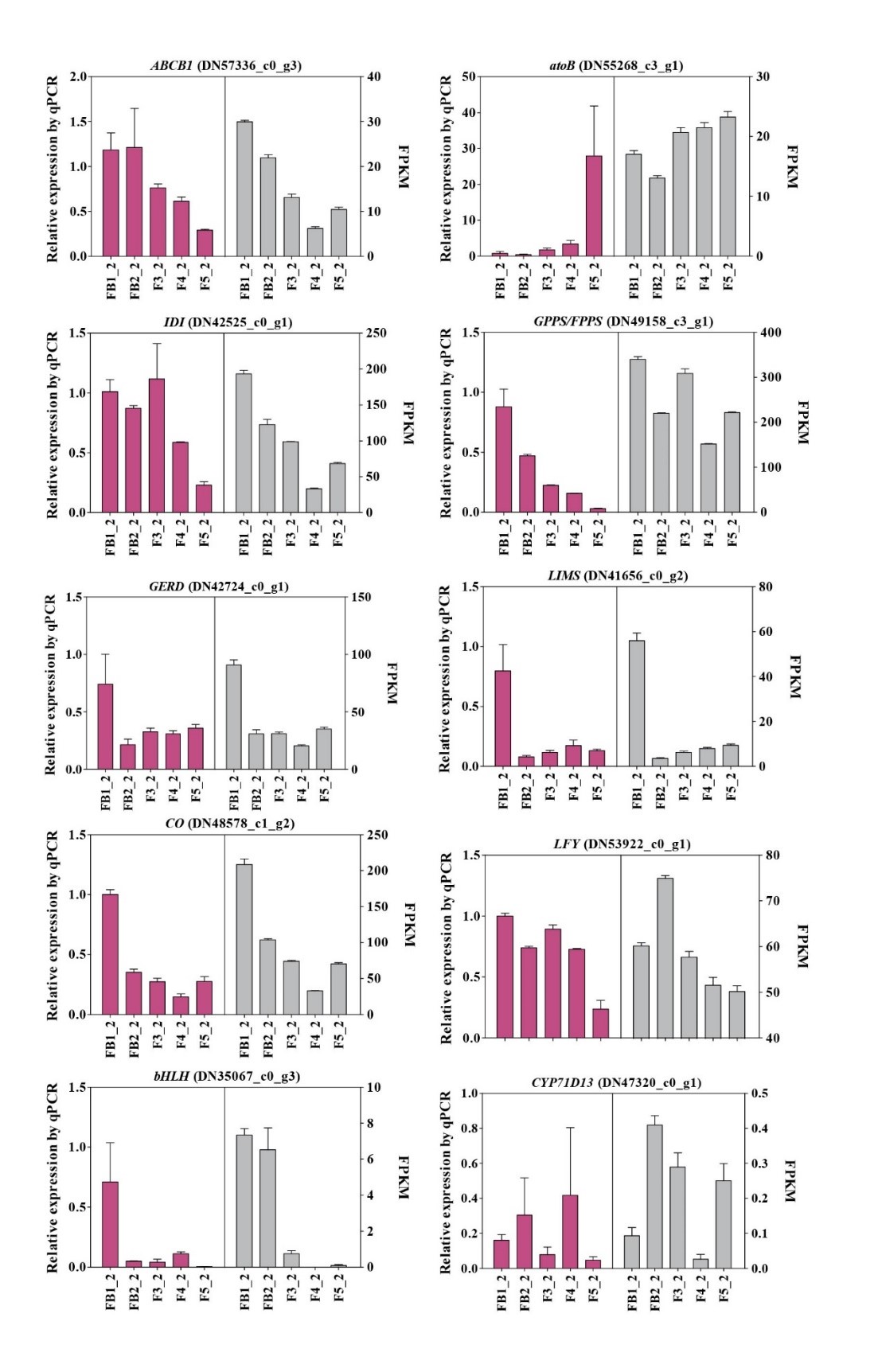

Supplement: Supplementary file 11 — Figure S11. Expression patterns of eight genes during different developmental stages of second-axis flower as verified by qRT-PCR. (JPG 259 kb) [file 12870_2019_1908_MOESM11_ESM.jpg]

## Slide 1
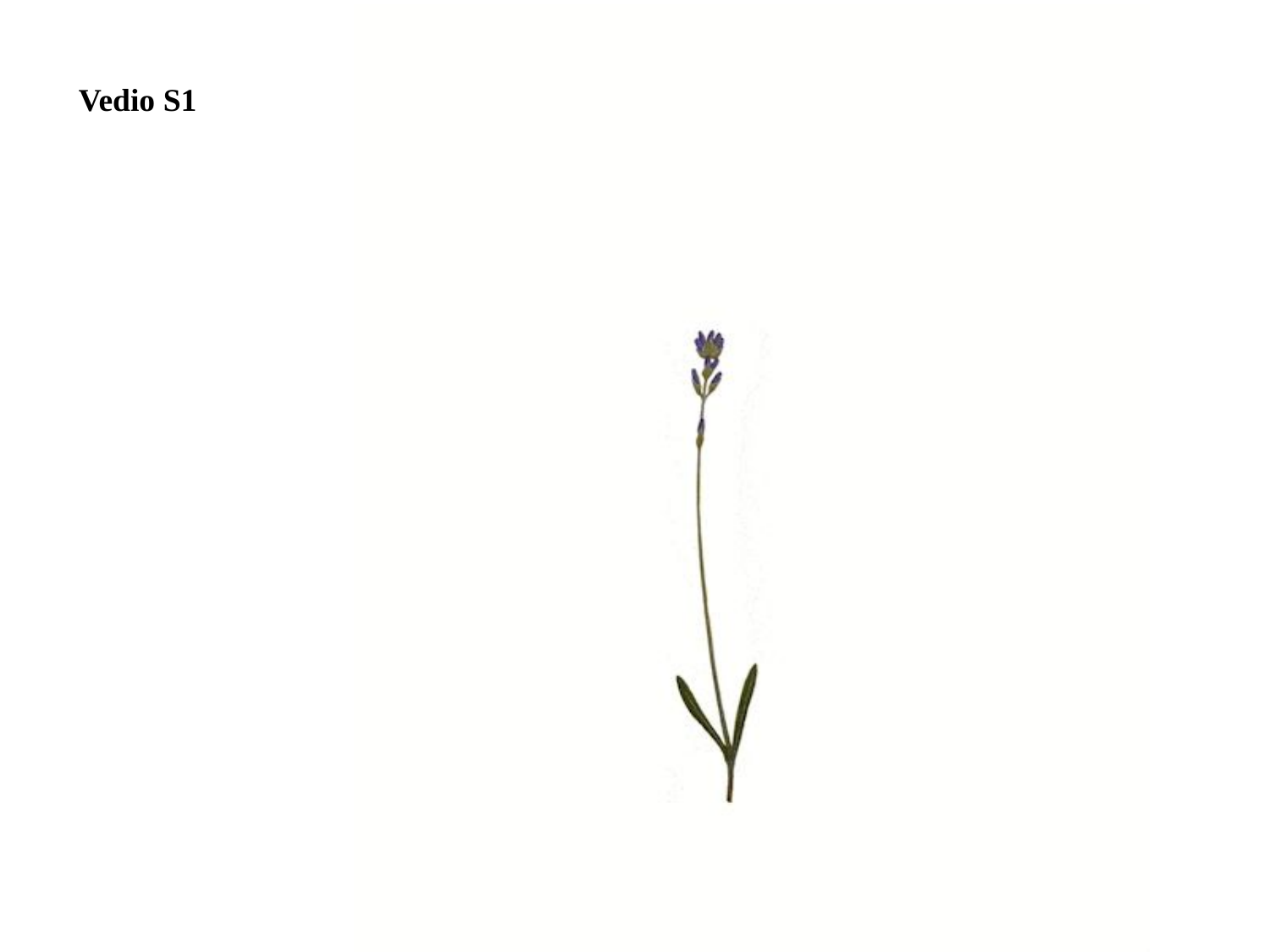

Vedio S1

Supplement: Supplementary file 19 — Video S1. Animation schematically illustrates the sequence of blossom in lavender. (PPTX 5149 kb) [file 12870_2019_1908_MOESM19_ESM.pptx]
