# Supplementary material for: Time-series transcriptome provides insights into the gene regulation network involved in the volatile terpenoid metabolism during the flower development of lavender
Source: BMC Plant Biol. 2019 Jul 15;19:313. doi: 10.1186/s12870-019-1908-6 (PMC6632208; doi:10.1186/s12870-019-1908-6)
Supplement: Supplementary file 12 — Table S1. Previously cloned genes for biosynthesis of mono- and sesquiterpenoids in lavenders. (DOCX 13 kb) [file 12870_2019_1908_MOESM12_ESM.docx]

**Additional file 12: Table S1** Previously cloned genes for biosynthesis of mono- and sesquiterpenoids in lavenders.

| Accession no. | Main products | Designation |
| --- | --- | --- |
| ABB73044 | Limonene | LaLIMS |
| ABB73045 | Linalool | LaLINS |
| ABB73046 | Bergamotene | LaBERS |
| HQ404305 | β-phellandrene | LaβPHLS |
| DQ886904.1 | alcohol acyltransferase 1 | LaAAT1 |
| HQ404306 | terpene synthase-like | LaTPS-I |
| DQ886905.1 | alcohol acyltransferase 2 | LaAAT2 |
| JX630156.1 | farnesyl diphosphate synthase | LaFPPS |
| JX630155.1 | hydroxymethylglutaryl-CoA reductase | LaHMGR |
| JX630153.1 | 4-hydroxy-3-methylbut-2-enyl diphosphate reductase | LaHDR |
| JX630152.1 | 4-hydroxy-3-methylbut-2-enyl diphosphate synthase | LaHDS |
| JX630151.1 | 1-deoxy-D-xylulose 5-phosphate reductoisomerase | LaDXR |
| JX630150.1 | 1-deoxy-D-xylulose 5-phosphate synthase | LaDXS2 |
| JX630149.1 | deoxyxylulose synthase | LaDXS1 |
| JX401284.1 | germacrene-D synthase | LaGERDS |
