# Supplementary material for: Time-series transcriptome provides insights into the gene regulation network involved in the volatile terpenoid metabolism during the flower development of lavender
Source: BMC Plant Biol. 2019 Jul 15;19:313. doi: 10.1186/s12870-019-1908-6 (PMC6632208; doi:10.1186/s12870-019-1908-6)
Supplement: Supplementary file 13 — Table S2. Contents of major mono- and sesquiterpenoids identified in EOs of lavender. (DOCX 86 kb) [file 12870_2019_1908_MOESM13_ESM.docx]

**Additional file 13: Table S2** Contents of major mono- and sesquiterpenoids identified in EOs of lavender. RT, Retention Time.

|  |  | Compounds | Formula | RT | FB0 | FB1_1 | FB2_1 | F3_1 | F4_1 | F5_1 | FB1_2 | FB2_1 | F3_2 | F4_2 | F5_2 |
| --- | --- | --- | --- | --- | --- | --- | --- | --- | --- | --- | --- | --- | --- | --- | --- |
| Monoterpenoids |  | *β*-thujene | C10H16 | 8.871 | 0.3106 | 0 | 0 | 0 | 0 | 0 | 0 | 0 | 0 | 0 | 0 |
|  |  | *α-*pinene | C10H16 | 9.081 | 1.3872 | 0 | 0 | 0 | 0 | 0 | 0 | 0 | 0 | 0 | 0 |
|  |  | camphene | C10H16 | 9.584 | 0.5544 | 0 | 0 | 0 | 0 | 0 | 0 | 0 | 0 | 0 | 0 |
|  |  | sabinene | C10H16 | 10.525 | 0.4691 | 0 | 0 | 0 | 0 | 0 | 0 | 0 | 0 | 0 | 0 |
|  |  | *β*-pinene | C10H16 | 10.601 | 1.7442 | 0.03 | 0 | 0 | 0 | 0 | 0.085 | 0.1 | 0.05 | 0 | 0.0095 |
|  |  | 1-octen-3-ol | C8H16O | 10.85 | 0.0672 | 0.05 | 0.1 | 0.1 | 0.2 | 0.2 | 0.096 | 0.1 | 0.19 | 0.08 | 0.1868 |
|  |  | *β*-myrcene | C10H16 | 11.079 | 1.8493 | 0.3 | 0.3 | 0.2 | 0.6 | 0.8 | 0.387 | 0.4 | 0.53 | 0.18 | 0.3278 |
|  |  | *α*-phellandrene | C10H16 | 11.651 | 0.3482 | 0 | 0 | 0 | 0 | 0 | 0.017 | 0 | 0 | 0 | 0.0066 |
|  |  | 3-carene | C10H16 | 11.855 | 3.8335 | 0.16 | 0.3 | 0 | 0.1 | 0.1 | 0.362 | 0.2 | 0.1 | 0.02 | 0.0482 |
|  |  | (+)-4-carene | C10H16 | 14.8 | 0.0291 | 0 | 0.1 | 0.1 | 0.3 | 0.3 | 0 | 0.1 | 0.24 | 0.1 | 0.2706 |
|  |  | o-cymene | C10H14 | 12.421 | 3.7242 | 0.12 | 0.1 | 0.1 | 0.5 | 0.6 | 0.558 | 0.3 | 0.44 | 0.14 | 0.3718 |
|  |  | (+)-(R)-limonene | C10H16 | 12.548 | 17 | 1.17 | 2.6 | 0.1 | 0.2 | 0.3 | 2.796 | 0.7 | 0.37 | 0.21 | 0.1067 |
|  |  | cineole | C10H18O | 12.618 | 0.489 | 0.55 | 0.4 | 0.1 | 0.4 | 0.4 | 0.57 | 0.3 | 0.37 | 0.11 | 0.2173 |
|  |  | *β*-trans-ocimene | C10H16 | 13.019 | 20.566 | 2.75 | 4 | 1.4 | 2.2 | 2.5 | 4.405 | 4.6 | 2.6 | 1.41 | 2.0659 |
|  |  | *β*-cis-ocimene | C10H16 | 13.356 | 4.4715 | 0.42 | 0.6 | 0.6 | 1 | 1.2 | 0.669 | 0.8 | 0.79 | 0.7 | 1.0063 |
|  |  | terpinolene | C10H16 | 14.788 | 0.5581 | 0.09 | 0.2 | 0.1 | 0.1 | 0.2 | 0.14 | 0.3 | 0.25 | 0.22 | 0.2281 |
|  |  | linalool | C10H18O | 15.258 | 2.1843 | 30.4 | 38 | 31 | 44 | 44 | 17.06 | 33 | 40.4 | 31.7 | 38.117 |
|  |  | 1-octenyl acetate | C10H18O2 | 15.748 | 0.4262 | 1.33 | 0.8 | 1.2 | 2.1 | 1.9 | 1.1 | 1.4 | 2 | 1.51 | 1.7953 |
|  |  | (E)-p-2-menthen-1-ol | C10H18O | 16.003 | 0.1932 | 0.07 | 0.1 | 0 | 0 | 0 | 0.139 | 0.1 | 0.06 | 0.02 | 0.0133 |
|  |  | allo-ocimene | C10H16 | 16.327 | 3.1152 | 0.42 | 0.3 | 0.2 | 0.3 | 0.4 | 0.6 | 0.7 | 0.37 | 0.22 | 0.3087 |
|  |  | (-)-camphor | C10H16O | 16.817 | 0.1905 | 0.14 | 0.2 | 0.1 | 0.2 | 0.1 | 0.196 | 0.1 | 0.12 | 0.08 | 0.1067 |
|  |  | endo-borneol | C10H18O | 17.6 | 1.5458 | 1.51 | 0.8 | 0.5 | 0.6 | 0.6 | 2.041 | 0.7 | 0.55 | 0.44 | 0.4498 |
|  |  | (±)-lavandulol | C10H18O | 17.714 | 1.5353 | 1.34 | 1.2 | 1.1 | 1.4 | 1.6 | 1.723 | 0.9 | 0.97 | 1.35 | 1.3646 |
|  |  | terpinen-4-ol | C10H18O | 18.039 | 0.2862 | 0.28 | 0.4 | 0.6 | 1 | 1.1 | 0.343 | 0.3 | 0.63 | 0.76 | 0.9987 |
|  |  | p-cymen-8-ol | C10H14O | 18.261 | 0.115 | 0.1 | 0.1 | 0.1 | 0 | 0.1 | 0.155 | 0.1 | 0.06 | 0.05 | 0.0498 |
|  |  | cryptone | C9H14O | 18.35 | 0.9698 | 0.77 | 0.7 | 0.3 | 0.2 | 0.1 | 0.971 | 0.7 | 0.46 | 0.25 | 0.1601 |
|  |  | *α*-terpineol | C10H18O | 19.528 | 0.6169 | 4.17 | 5.2 | 3.9 | 4.8 | 4.4 | 2.821 | 4.4 | 4.72 | 4.3 | 2.9312 |
|  |  | n-hexyl butanoate | C10H18O | 18.637 | 0 | 0.03 | 0.2 | 0.1 | 0.3 | 0.4 | 0.037 | 0.1 | 0.22 | 0.31 | 0.3583 |
|  |  | myrtenol | C10H16O | 18.719 | 0.1479 | 0.1 | 0.1 | 0 | 0 | 0 | 0.147 | 0.1 | 0.07 | 0.02 | 0.012 |
|  |  | cis-piperitol | C10H18O | 19.158 | 0.1183 | 0.03 | 0 | 0 | 0 | 0 | 0.122 | 0.1 | 0.01 | 0.02 | 0.018 |
|  |  | cis-geraniol | C10H18O | 19.896 | 0.0546 | 0.47 | 0.1 | 0.7 | 0.7 | 0.7 | 0.398 | 0.7 | 0.7 | 0.69 | 0.4269 |
|  |  | cumaldehyde | C10H12O | 20.272 | 0.2394 | 0.1 | 0.1 | 0.1 | 0.1 | 0 | 0.227 | 0.1 | 0.13 | 0.09 | 0.0243 |
|  |  | (-)-carvone | C10H14O | 20.444 | 0.0926 | 0.06 | 0.1 | 0.1 | 0.1 | 0 | 0.102 | 0.1 | 0.07 | 0.01 | 0.0334 |
|  |  | teresantalol | C10H16O | 29.312 | 0.2304 | 0.1 | 0.1 | 0.1 | 0.1 | 0.1 | 0.137 | 0.1 | 0.06 | 0.08 | 0.0492 |
| Sesquiterpenoids |  | linalyl acetate | C12H20O2 | 20.851 | 2.0155 | 23.4 | 21 | 32 | 21 | 19 | 27.65 | 26 | 22.5 | 32 | 31.369 |
|  |  | dihydrocarvyl acetate | C12H20O2 | 21.659 | 0.1115 | 0.14 | 0.1 | 0.1 | 0.1 | 0.1 | 0.122 | 0.1 | 0.1 | 0.08 | 0.0115 |
|  |  | bornyl acetate | C12H20O2 | 21.856 | 0.7283 | 0.59 | 0.3 | 0.2 | 0.2 | 0.2 | 0.783 | 0.3 | 0.19 | 0.16 | 0.1144 |
|  |  | lavandulyl acetate | C12H20O2 | 22.104 | 15.235 | 21.9 | 16 | 16 | 12 | 12 | 24.46 | 16 | 13.9 | 15 | 12.007 |
|  |  | nerol acetate | C12H20O2 | 24.496 | 0.3254 | 1.28 | 1.5 | 1.4 | 1.4 | 1.4 | 1.037 | 1.2 | 1.36 | 1.4 | 0.8119 |
|  |  | geranyl acetate | C12H20O2 | 25.113 | 0.71 | 1.86 | 2.2 | 3.2 | 2.7 | 2.9 | 1.706 | 2.4 | 2.6 | 2.78 | 1.5895 |
|  |  | copaene | C15H24 | 24.846 | 0.1532 | 0.04 | 0 | 0 | 0 | 0 | 0.063 | 0 | 0.01 | 0.01 | 0.0191 |
|  |  | *α*-bergamotene | C15H24 | 26.112 | 0.2519 | 0.13 | 0 | 0.1 | 0 | 0 | 0.205 | 0 | 0.02 | 0.02 | 0.0142 |
|  |  | *β*-caryophyllene | C15H24 | 26.239 | 3.9096 | 1.84 | 1.2 | 1.6 | 0.8 | 1.3 | 2.68 | 1.3 | 0.8 | 1.66 | 1.0063 |
|  |  | epi-*β*-santalene | C15H24 | 27.111 | 0.1493 | 0.02 | 0 | 0 | 0 | 0 | 0.077 | 0 | 0.02 | 0.01 | 0.009 |
|  |  | E-*β*-farnesene | C15H24 | 28.275 | 0.7041 | 0.22 | 0.1 | 0.3 | 0.2 | 0.5 | 0.343 | 0.2 | 0.17 | 0.48 | 0.3697 |
|  |  | germacrene D | C15H24 | 28.16 | 1.443 | 0.21 | 0 | 0.4 | 0.1 | 0.2 | 0.437 | 0.3 | 0.1 | 0.34 | 0.1906 |
|  |  | cis-*β*-famesene | C15H24 | 27.41 | 0.27 | 0.03 | 0 | 0 | 0 | 0 | 0.067 | 0 | 0.02 | 0.03 | 0.0188 |
|  |  | *γ*-cadinene | C15H24 | 29.159 | 0.5011 | 0.03 | 0 | 0 | 0 | 0 | 0.051 | 0 | 0.03 | 0.01 | 0.0046 |
|  |  | epi-cubebol | C15H26O | 29.439 | 0.16 | 0.07 | 0 | 0 | 0 | 0 | 0.077 | 0 | 0.02 | 0.01 | 0.0044 |
|  |  | farnesene epoxide | C15H24O | 29.624 | 0.1098 | 0.05 | 0 | 0 | 0 | 0 | 0.095 | 0 | 0.02 | 0.03 | 0.0044 |
|  |  | cis-sesquisabinene hydrate | C15H26O | 30.33 | 0.14 | 0.02 | 0 | 0 | 0 | 0 | 0.017 | 0 | 0.02 | 0.01 | 0.0101 |
|  |  | caryophyllene oxide | C15H24O | 31.195 | 0.4129 | 0.51 | 0.5 | 0.5 | 0.3 | 0.3 | 0.588 | 0.3 | 0.23 | 0.4 | 0.2287 |
|  |  | epi-cubenol | C15H26O | 29.159 | 0.2427 | 0.01 | 0 | 0 | 0 | 0 | 0.136 | 0 | 0 | 0 | 0 |
|  |  | *α*-epi-cadinol | C15H26O | 32.843 | 2.2159 | 0.2 | 0 | 0.1 | 0 | 0 | 0.233 | 0.1 | 0.05 | 0.07 | 0.0246 |
|  |  | ledol | C15H26O | 33.11 | 0.13 | 0.02 | 0 | 0 | 0 | 0 | 0.024 | 0.1 | 0.01 | 0.02 | 0.0048 |
|  |  | allohimachalol | C15H26O | 33.689 | 0.3 | 0.2 | 0 | 0.1 | 0 | 0 | 0.193 | 0 | 0.04 | 0.07 | 0.0268 |
| Total (%) | | | | | 99.682 | 99.9 | 100 | 100 | 100 | 100 | 99.45 | 100 | 99.8 | 99.8 | 99.902 |
