# Supplementary material for: Time-series transcriptome provides insights into the gene regulation network involved in the volatile terpenoid metabolism during the flower development of lavender
Source: BMC Plant Biol. 2019 Jul 15;19:313. doi: 10.1186/s12870-019-1908-6 (PMC6632208; doi:10.1186/s12870-019-1908-6)
Supplement: Supplementary file 14 — Table S3. Information of Illumina reads from 33 libraries. (DOCX 16 kb) [file 12870_2019_1908_MOESM14_ESM.docx]

**Additional file 14: Table S3** Information of Illumina reads from 33 libraries.

| Sample | Raw Reads | Clean reads | Clean bases | Error (%) | Q20 (%) | Q30 (%) | GC  (%) | Total mapped | Mapping rate |
| --- | --- | --- | --- | --- | --- | --- | --- | --- | --- |
| FB0_1 | 50688110 | 49482500 | 7.42G | 0.01 | 97.79 | 94.37 | 47.37 | 39206330 | 79.23% |
| FB0_2 | 47683378 | 46492922 | 6.97G | 0.01 | 97.87 | 94.54 | 47.51 | 37147590 | 79.90% |
| FB0_3 | 55077914 | 53873980 | 8.08G | 0.01 | 97.67 | 94.08 | 47.01 | 42707896 | 79.27% |
| FB1_1_1 | 57113424 | 55888828 | 8.38G | 0.01 | 97.69 | 94.13 | 47.03 | 44353810 | 79.36% |
| FB1_1_2 | 56594214 | 55122056 | 8.27G | 0.02 | 97.03 | 92.68 | 47.17 | 43454756 | 78.83% |
| FB1_1_3 | 54791988 | 53530958 | 8.03G | 0.01 | 97.67 | 94.1 | 47.31 | 42474060 | 79.34% |
| FB1_2_1 | 54424302 | 53186758 | 7.98G | 0.01 | 97.59 | 93.91 | 47.31 | 41643662 | 78.30% |
| FB1_2_2 | 55511196 | 53997650 | 8.1G | 0.02 | 96.89 | 92.17 | 46.86 | 42498542 | 78.70% |
| FB1_2_3 | 43311560 | 41699382 | 6.25G | 0.02 | 96.59 | 91.54 | 46.87 | 32688498 | 78.39% |
| FB2_1_1 | 50203654 | 49037880 | 7.36G | 0.01 | 97.66 | 94.06 | 47.44 | 38601346 | 78.72% |
| FB2_1_2 | 53076770 | 51825938 | 7.77G | 0.01 | 97.71 | 94.17 | 47.2 | 41272246 | 79.64% |
| FB2_1_3 | 61453596 | 60121982 | 9.02G | 0.01 | 97.72 | 94.2 | 47.31 | 47570412 | 79.12% |
| FB2_2_1 | 55623658 | 54346718 | 8.15G | 0.01 | 97.72 | 94.21 | 47.39 | 43249512 | 79.58% |
| FB2_2_2 | 54166610 | 52918686 | 7.94G | 0.01 | 97.76 | 94.29 | 47.15 | 42352028 | 80.03% |
| FB2_2_3 | 52963788 | 51798514 | 7.77G | 0.01 | 97.69 | 94.13 | 47.06 | 41206636 | 79.55% |
| F3_1_1 | 64314406 | 62923582 | 9.44G | 0.01 | 97.64 | 94.01 | 47.09 | 49629748 | 78.87% |
| F3_1_2 | 54677182 | 53472236 | 8.02G | 0.01 | 97.61 | 93.94 | 47.19 | 41935104 | 78.42% |
| F3_1_3 | 45604792 | 44135554 | 6.62G | 0.02 | 96.06 | 90.57 | 47.06 | 34162340 | 77.40% |
| F3_2_1 | 56728812 | 55419532 | 8.31G | 0.01 | 97.63 | 94.02 | 47.15 | 43511568 | 78.51% |
| F3_2_2 | 55760080 | 54433776 | 8.17G | 0.01 | 97.63 | 94 | 47.34 | 42846420 | 78.71% |
| F3_2_3 | 64743930 | 63331240 | 9.5G | 0.01 | 97.58 | 93.89 | 47.21 | 49578564 | 78.28% |
| F4_1_1 | 50307196 | 49252076 | 7.39G | 0.01 | 97.39 | 93.51 | 46.99 | 38305096 | 77.77% |
| F4_1_2 | 55013130 | 53845182 | 8.08G | 0.01 | 97.46 | 93.65 | 46.87 | 41954346 | 77.92% |
| F4_1_3 | 56632320 | 55419596 | 8.31G | 0.01 | 97.43 | 93.59 | 47 | 43223816 | 77.99% |
| F4_2_1 | 56556920 | 55371232 | 8.31G | 0.01 | 97.41 | 93.53 | 46.99 | 43626156 | 78.79% |
| F4_2_2 | 48560272 | 47549298 | 7.13G | 0.01 | 97.5 | 93.71 | 46.66 | 37773902 | 79.44% |
| F4_2_3 | 47806910 | 46654332 | 7G | 0.01 | 97.51 | 93.76 | 46.72 | 36762430 | 78.80% |
| F5_1_1 | 50885162 | 49692040 | 7.45G | 0.02 | 97.18 | 92.98 | 47.19 | 39305058 | 79.10% |
| F5_1_2 | 56738628 | 55346394 | 8.3G | 0.01 | 97.37 | 93.44 | 47.03 | 43843680 | 79.22% |
| F5_1_3 | 47126184 | 46094404 | 6.91G | 0.01 | 97.37 | 93.46 | 47.27 | 36226826 | 78.59% |
| F5_2_1 | 57864570 | 56464974 | 8.47G | 0.01 | 97.42 | 93.55 | 46.98 | 44196162 | 78.27% |
| F5_2_2 | 58817044 | 57553286 | 8.63G | 0.02 | 97.28 | 93.26 | 46.87 | 45218872 | 78.57% |
| F5_2_3 | 48037348 | 47006200 | 7.05G | 0.01 | 97.4 | 93.52 | 47.02 | 36880572 | 78.46% |
