# Supplementary material for: Time-series transcriptome provides insights into the gene regulation network involved in the volatile terpenoid metabolism during the flower development of lavender
Source: BMC Plant Biol. 2019 Jul 15;19:313. doi: 10.1186/s12870-019-1908-6 (PMC6632208; doi:10.1186/s12870-019-1908-6)
Supplement: Supplementary file 15 — Table S4. Annotation of all unigenes based on seven databases. (DOCX 13 kb) [file 12870_2019_1908_MOESM15_ESM.docx]

**Additional file 15: Table S4** Annotation of all unigenes based on seven databases.

|  | Number of Unigenes | Percentage (%) |
| --- | --- | --- |
| NR | 61440 | 38.55 |
| NT | 44134 | 27.69 |
| KEGG | 22483 | 14.11 |
| SwissProt | 45021 | 28.25 |
| PFAM | 43005 | 26.98 |
| GO | 43539 | 27.32 |
| KOG | 12490 | 7.83 |
| Annotated in all Databases | 6599 | 4.14 |
| Annotated in at least one Database | 78464 | 49.24 |
| Total Unigenes | 159337 | 100 |
