# Supplementary material for: Time-series transcriptome provides insights into the gene regulation network involved in the volatile terpenoid metabolism during the flower development of lavender
Source: BMC Plant Biol. 2019 Jul 15;19:313. doi: 10.1186/s12870-019-1908-6 (PMC6632208; doi:10.1186/s12870-019-1908-6)
Supplement: Supplementary file 16 — Table S5. Summary of putative genes of CYP450s family in lavender. (DOCX 15 kb) [file 12870_2019_1908_MOESM16_ESM.docx]

**Additional file 16: Table S5** Summary of putative genes of CYP450s family in lavender.

| Name | No. | unigenes |
| --- | --- | --- |
| CYP26A | 4 | DN51200_c1_g3; DN62140_c0_g1; DN2141_c0_g1; DN53256_c0_g6 |
| CYP701A40 | 1 | DN45294_c1_g1 |
| CYP704C1 | 4 | DN54251_c1_g1; DN54251_c1_g2; DN52947_c2_g1; DN57308_c4_g11 |
| CYP707A1 | 1 | DN53370_c2_g1 |
| CYP707A2 | 1 | DN51009_c5_g2 |
| CYP707A3 | 1 | DN45505_c4_g4 |
| CYP711A1 | 1 | DN56628_c1_g2 |
| CYP714A2 | 1 | DN49894_c0_g1 |
| CYP714C2 | 8 | DN48711_c7_g3; DN54490_c0_g3; DN56369_c2_g6; DN47909_c4_g3; DN56369_c2_g1; DN56369_c2_g2; DN56369_c2_g3; DN56369_c2_g7 |
| CYP716A89 | 1 | DN48439_c0_g3 |
| CYP71A1 | 3 | DN50254_c3_g6; DN40252_c0_g3; DN45282_c1_g1 |
| CYP71A6 | 1 | DN39854_c1_g2 |
| CYP71A8 | 2 | DN41273_c0_g1; DN22690_c0_g1 |
| CYP71D10 | 1 | DN45475_c4_g2 |
| CYP71D13 | 1 | DN47320_c0_g1 |
| CYP71D55 | 1 | DN46629_c0_g1 |
| CYP725A1 | 1 | DN51921_c0_g2 |
| CYP72A219 | 8 | DN48660_c1_g4; DN48657_c3_g1; DN55623_c1_g1; DN45009_c1_g5; DN45009_c1_g6; DN46458_c0_g1; DN56960_c1_g1; DN56960_c2_g5 |
| CYP736A12 | 6 | DN53587_c1_g3; DN51606_c0_g1; DN46598_c3_g2; DN45649_c0_g3; DN53587_c1_g2; DN45649_c0_g2 |
| CYP73A4 | 2 | DN41887_c0_g5; DN51454_c1_g1 |
| CYP749A22 | 2 | DN50593_c1_g4; DN51895_c0_g3 |
| CYP74A | 1 | DN45486_c1_g1 |
| CYP75A2 | 1 | DN50266_c0_g1 |
| CYP75A3 | 2 | DN51437_c0_g1; DN51437_c0_g3 |
| CYP75B1 | 1 | DN46460_c2_g1 |
| CYP75B2 | 1 | DN45491_c0_g2 |
| CYP76A2 | 1 | DN52126_c1_g3 |
| CYP76AH1 | 3 | DN52877_c1_g1; DN55169_c0_g1; DN50237_c0_g1 |
| CYP76B1 | 1 | DN51691_c2_g7 |
| CYP76B6 | 1 | DN51990_c0_g1 |
| CYP77A1 | 1 | DN53204_c1_g1 |
| CYP77A2 | 1 | DN43602_c0_g1 |
| CYP77A3 | 1 | DN49104_c0_g1 |
| CYP78A7 | 1 | DN46851_c0_g1 |
| CYP78A9 | 1 | DN40623_c0_g1 |
| CYP79A2 | 1 | DN36017_c0_g2 |
| CYP80B2 | 1 | DN42827_c1_g2 |
| CYP81D11 | 1 | DN47603_c0_g1 |
| CYP81E8 | 3 | DN52484_c2_g1; DN44532_c0_g1; DN48348_c3_g1 |
| CYP82A3 | 2 | DN36861_c0_g3; DN44205_c0_g1 |
| CYP82C2 | 1 | DN41762_c1_g2 |
| CYP82G1 | 1 | DN77382_c0_g1 |
| CYP83B1 | 1 | DN54433_c0_g2 |
| CYP84A1 | 1 | DN39855_c0_g1 |
| CYP85A1 | 1 | DN54737_c1_g2 |
| CYP86A1 | 1 | DN49176_c0_g1 |
| CYP86A2 | 2 | DN52406_c4_g2; DN52406_c4_g4 |
| CYP86A7 | 1 | DN52406_c4_g3 |
| CYP86B1 | 1 | DN34299_c1_g1 |
| CYP88A52 | 1 | DN51152_c2_g2 |
| CYP89A2 | 1 | DN56491_c1_g2 |
| CYP90A1 | 1 | DN52420_c1_g1 |
| CYP90B1 | 1 | DN51763_c0_g2 |
| CYP90C19 | 1 | DN50971_c1_g3 |
| CYP90D1 | 1 | DN40451_c0_g1 |
| CYP93B1 | 1 | DN52410_c0_g3 |
| CYP94A1 | 1 | DN45700_c0_g1 |
| CYP94B1 | 1 | DN30910_c0_g2 |
| CYP94B3 | 4 | DN30910_c0_g1; DN49586_c0_g1; DN49586_c0_g2; DN53566_c0_g1 |
| CYP94C1 | 2 | DN45560_c0_g1; DN49918_c2_g1 |
| CYP94D47 | 1 | DN55874_c0_g1 |
| CYP96A15 | 3 | DN50838_c0_g4; DN29486_c0_g1; DN50838_c0_g2 |
| CYP98A3 | 2 | DN48869_c1_g2; DN49166_c1_g3 |
