# Supplementary material for: Time-series transcriptome provides insights into the gene regulation network involved in the volatile terpenoid metabolism during the flower development of lavender
Source: BMC Plant Biol. 2019 Jul 15;19:313. doi: 10.1186/s12870-019-1908-6 (PMC6632208; doi:10.1186/s12870-019-1908-6)
Supplement: Supplementary file 17 — Table S6. Summary of putative terpenoid transporters in lavender. (DOCX 12 kb) [file 12870_2019_1908_MOESM17_ESM.docx]

**Additional file 17: Table S6** Summary of putative terpenoid transporters in lavender.

| transporter | No. | unigenes |
| --- | --- | --- |
| ABC | 17 | DN50774_c2_g7; DN57157_c0_g1; DN46936_c0_g1; DN56065_c2_g2; DN55900_c2_g4; DN57336_c0_g4; DN42037_c0_g1; DN43846_c0_g1; DN53017_c0_g8; DN55335_c6_g4; DN57336_c0_g3; DN57073_c1_g1; DN53986_c0_g4; DN56924_c0_g1; DN41691_c0_g2; DN52839_c1_g1; DN54335_c2_g1 |
| BOR2 | 3 | DN46919_c0_g2; DN46919_c0_g1; DN54691_c1_g2 |
| BOR4 | 2 | DN57111_c1_g1; DN54691_c1_g3 |
| LTP | 8 | DN47207_c3_g2; DN48902_c0_g1; DN38886_c0_g2; DN47207_c1_g2; DN39526_c0_g1; DN39526_c0_g2; DN39526_c0_g3; DN51591_c1_g2 |
| DLAT | 1 | DN56096_c1_g3 |
| NPF3.1 | 1 | DN49927_c2_g1 |
