# Supplementary material for: Time-series transcriptome provides insights into the gene regulation network involved in the volatile terpenoid metabolism during the flower development of lavender
Source: BMC Plant Biol. 2019 Jul 15;19:313. doi: 10.1186/s12870-019-1908-6 (PMC6632208; doi:10.1186/s12870-019-1908-6)
Supplement: Supplementary file 18 — Table S7. Gene-specific primer pairs used for qRT-PCR. (DOCX 13 kb) [file 12870_2019_1908_MOESM18_ESM.docx]

**Additional file 18: Table S7** Gene-specific primer pairs used for qRT-PCR.

| Gene name | Forward primer (5’-3’) | Reverse primer (5’-3’) |
| --- | --- | --- |
| *atoB* (DN55268_c3_g1) | AGATTCACAAGAAGCGCGGA | GCAAATGAGGGCGGTTTGTT |
| *GPPS/FPPS* (DN49158_c3_g1) | GAATGCCACTTGATGCGTGG | ACGCCCTCCACTAAAGCATC |
| *LIMS* (DN41656_c0_g2) | GGCGGAGATGATTTTGCGAG | GAAATCGCCCTCCTCGTTCT |
| *CYP71D13* (DN47320_c0_g1) | TGAGGATGCGCAGTAAGCTC | GGCCTTCTGCATTGCTTCTG |
| *ABCB1* (DN57336_c0_g3) | ACCGATGCTAGAACTGGGGA | CAAAGGCGATCCCCGGAATA |
| *CO* (DN48578_c1_g2) | GTCCTGTCGTTGAGGGGTTC | GCTTCTCTGTCCATTCCCGA |
| *LFY* (DN53922_c0_g1) | TACGTGTTGTACGAGTCGGC | GGAAATGGCGTTGCATTGGT |
| *bHLH* (DN35067_c0_g3) | TCCAGAAGCCAGTCGTTGAG | TTCGCGGTTCACCGAAGAAG |
| *18S rRNA* | AACGACTCTCGGCAACGGATA | GCGTTCAAAGACTCGATGGT |
| *actin* | TGTGGATTGCCAAGGCAGAGT | AATGAGCAGGCAGCAACAGCA |
